# Non-invasive molecular surveillance of drug-resistant bacterial and fungal pathogens in severe ICU pneumonia: a comparative study of nasopharyngeal swabs and BALF

**DOI:** 10.3389/fmicb.2026.1794266

**Published:** 2026-04-22

**Authors:** Hua Li, Yicheng Yang, Chun Lin, Daoyong Huang, Mingyue Lin, Ziting Wu, Haidan Lin, Xiao Zhan, Zufu Cheng, Youshe Lu, Ling Ouyang

**Affiliations:** 1 Liwan Central Hospital of Guangzhou, Guangzhou, Guangdong, China; 2 Guangzhou Labway Clinical Laboratory Co., Ltd, Guangzhou, Guangdong, China

**Keywords:** bronchoalveolar lavage fluid, drug-resistant infections, ICU, immunocompromised hosts, nasopharyngeal swab, severe pneumonia, targeted next-generation sequencing

## Abstract

**Background:**

Drug-resistant bacterial and fungal infections represent a major cause of morbidity and mortality among critically ill and immunocompromised patients with severe pneumonia. Molecular diagnostics using bronchoalveolar lavage fluid (BALF) are considered the reference standard for pathogen identification but are invasive and often contraindicated in unstable intensive care unit (ICU) patients. Safer non-invasive sampling strategies that maintain diagnostic reliabilityfor clinically relevant and potentially drug-resistant pathogens are therefore urgently needed.

**Methods:**

In this prospective cohort study conducted between January 1 and July 30, 2024, paired nasopharyngeal swabs (NPS) and BALF samples were collected from 105 ICU patients with severe pneumonia. Pathogen detection was performed using targeted next-generation sequencing (tNGS) with a focus on clinically significant bacterial, viral, and fungal pathogens commonly associated with antimicrobial resistance. Concordance between NPS and BALF was evaluated using positive percent agreement (PPA) and negative percent agreement (NPA), with BALF serving as the reference standard. Longitudinal two-phase sampling was performed in 74 patients to assess the capacity of NPS to monitor temporal changes in pathogen profiles.

**Results:**

Compared with BALF, NPS demonstrated high concordance for major bacterial pathogens frequently associated with drug resistance, including *Acinetobacter baumannii*, *Staphylococcus aureus*, and *Escherichia coli*, with PPA ≥ 80.0% and overall detection rates ≥90.0%. NPA exceeded 95.0% for most pathogens, indicating a low false-positive rate. Viral detection showed moderate sensitivity (PPA ≤ 80.0%), while fungal concordance was variable, ranging from 0.0% for *Aspergillus flavus* to 50.0% for *Pneumocystis jirovecii*. Longitudinal analyses revealed limited utility of NPS for monitoring dynamic pathogen changes over time, with higher agreement observed only for *Staphylococcus aureus* (72.7%), *Acinetobacter baumannii* (62.5%), and fungal pathogens (75.0%).

**Conclusion:**

Nasopharyngeal swab–based tNGS represents a feasible and less invasive approach for the early molecular identification of common bacterial pathogens, including those frequently implicated in drug-resistant infections, in patients with severe ICU pneumonia. While NPS may support early antimicrobial decision-making when BALF is not immediately available, its reduced sensitivity for fungal and viral pathogens and limited performance in longitudinal surveillance underscore the continued necessity of BALF for comprehensive infection assessment, particularly in immunocompromised and high-risk ICU populations.

## Introduction

Timely and accurate identification of causative pathogens in severe pneumonia is a critical determinant of clinical outcomes among critically ill patients (Zhang et al., 2024). Selection of the optimal specimen for etiological diagnosis requires careful consideration of diagnostic performance, laboratory feasibility, procedural safety, and cost–benefit balance (Messacar et al., 2017). In clinical practice, bronchoalveolar lavage fluid (BALF) has long been regarded as the reference standard for lower respiratory tract infection diagnostics, as it enables cytological assessment, molecular testing, rapid staining, and both qualitative and quantitative microbial cultures (Torres and Fernández-Barat, 2014). However, bronchoalveolar lavage (BAL) is a semi-invasive procedure, and procedure-related adverse events particularly hypoxemia, hemodynamic instability, and bronchospasm are not uncommon in intensive care unit (ICU) patients with severe pneumonia (Kamel et al., 2020). Moreover, BAL requires experienced bronchoscopists and advanced cardiopulmonary monitoring infrastructure, limiting its accessibility in resource-constrained or emergency settings. Alternative respiratory specimens, such as expectorated sputum, are frequently suboptimal. More than half of patients with severe pneumonia are unable to produce adequate sputum samples, and when available, diagnostic accuracy is often compromised by contamination with oropharyngeal flora, reducing specificity for true lower respiratory pathogens (Gadsby et al., 2016). In contrast, upper respiratory tract sampling using nasopharyngeal swabs (NPS) and oropharyngeal swabs (OPS) is non-invasive, readily accessible, and widely adopted for molecular diagnosis of viral pneumonia and atypical bacterial infections, including *Mycoplasma pneumoniae* and *Chlamydophila pneumoniae* (Murdoch, 2016). Nevertheless, for common bacterial pathogens such as *Streptococcus pneumoniae*, *Haemophilus influenzae*, and *Moraxella catarrhalis*, interpretation of NPS/OPS results remains controversial, as these organisms may colonize the upper respiratory tract as part of the normal microbiota. Several studies have assessed the diagnostic utility of NPS/OPS for lower respiratory tract infections (Nyawanda et al., 2019; Demars et al., 2022; Serigstad et al., 2023). Recent studies have further explored the application of molecular diagnostics, including targeted next-generation sequencing and multiplex PCR platforms, for pathogen identification in severe pneumonia. These approaches have demonstrated improved sensitivity for detecting bacterial and viral pathogens in respiratory samples and may facilitate earlier antimicrobial optimization in critically ill patients (Hattab et al., 2024; Zhang et al., 2024; Shang et al., 2025). Most were retrospective in design and employed heterogeneous diagnostic platforms ranging from pathogen-specific assays to multiplex syndromic panels. While consistently high negative percent agreement (NPA ≥ 98.0%) was reported, positive percent agreement (PPA) varied widely (58.0–86.0%), underscoring uncertainty regarding the reliability of upper respiratory specimens for pathogen confirmation (Hattab et al., 2024). In this prospective cohort study, we compared pathogen detection between paired NPS and BALF samples from ICU patients with severe pneumonia using targeted next-generation sequencing (tNGS). In addition to cross-sectional concordance, we implemented a two-phase longitudinal sampling strategy to assess the capacity of NPS to reflect temporal changes in pathogen profiles during the disease course. Importantly, this study was designed to address two key methodological concerns inherent to upper respiratory tract–based molecular diagnostics: colonization bias and sequencing depth–related variability. Colonization bias arises from the physiological presence of commensal and potentially pathogenic organisms in the upper airway, which may lead to false attribution of infection when highly sensitive molecular assays are applied. To mitigate this, BALF was used as the reference standard, and pathogen agreement metrics were interpreted in the context of organism-specific detection patterns. In parallel, the use of a standardized tNGS workflow with controlled library preparation and uniform sequencing depth enabled a robust comparison between specimen types, minimizing technical variability that could confound pathogen detection or longitudinal interpretation. By explicitly integrating these considerations, the present study provides a methodologically rigorous assessment of the clinical utility and limitations of NPS-based molecular diagnostics for severe pneumonia in critically ill patients, with direct implications for antimicrobial decision-making and stewardship in the ICU setting.

## Materials and methods

This study was conducted in accordance with the principles of the Declaration of Helsinki and complied with all applicable ethical guidelines and regulations. Ethical approval was obtained from the Medical Ethics Committee of Liwan Central Hospital of Guangzhou (approval number: 2024010902). Given the retrospective study design and the use of anonymized clinical data, the requirement for written informed consent was waived by the ethics committee.

### Study design and cohort selection

This single-center prospective cohort study was conducted at Liwan Central Hospital of Guangzhou, China, between January 1 and July 30, 2024. The study aimed to evaluate the diagnostic concordance between nasopharyngeal swabs (NPS) and bronchoalveolar lavage fluid (BALF) for pathogen identification in critically ill patients with severe pneumonia using targeted next-generation sequencing (tNGS). Consecutive adult patients (≥18 years) admitted to the intensive care unit (ICU) with suspected severe pneumonia were screened for eligibility. Severe pneumonia was defined according to the 2019 American Thoracic Society/Infectious Diseases Society of America (ATS/IDSA) guidelines and corresponding Chinese clinical practice guidelines (Torres et al., 2017; Cao et al., 2018; Metlay et al., 2019). Patients were classified as having severe pneumonia if they fulfilled at least one major criterion (respiratory failure requiring invasive mechanical ventilation or septic shock requiring vasopressor support) or three or more minor criteria, including respiratory rate ≥30 breaths/min, PaO₂/FiO₂ ≤ 250, multilobar pulmonary infiltrates, altered mental status, blood urea nitrogen ≥20 mg/dL, leukopenia (white blood cell count <4 × 10^9^/L), thrombocytopenia (platelet count <100 × 10^9^/L), hypothermia (<36.0 °C), or hypotension requiring aggressive fluid resuscitation. Radiological confirmation of pneumonia was mandatory and established by chest radiography or computed tomography demonstrating new or progressive pulmonary infiltrates consistent with lower respiratory tract infection. Patients were eligible for inclusion if diagnostic bronchoalveolar lavage was clinically indicated as part of routine ICU management and if paired NPS and BALF specimens could be obtained within the same diagnostic window. Because bronchoalveolar lavage was performed only when clinically indicated, the study population may represent a more severe subset of ICU pneumonia patients. Exclusion criteria were predefined to ensure patient safety and data quality and included: (1) severe hypoxemia or respiratory instability precluding safe bronchoscopy, (2) acute or unstable cardiovascular disease, including uncontrolled arrhythmia or shock, (3) coagulopathy or thrombocytopenia contraindicating bronchoscopy, (4) anatomical abnormalities of the airway preventing adequate sampling, and (5) refusal or inability to provide informed consent, when applicable. Demographic characteristics, comorbidities, severity indices, laboratory parameters, radiological findings, and antimicrobial exposure prior to sampling were systematically collected at enrollment. Patients who underwent repeat paired sampling during the course of ICU treatment were included in a predefined longitudinal subgroup to assess temporal concordance and pathogen dynamics. Because this study was designed as an exploratory prospective cohort investigation, a formal sample size calculation was not performed. The target sample size was determined based on the expected number of ICU patients with severe pneumonia undergoing clinically indicated bronchoalveolar lavage during the study period. A total of 105 patients were enrolled, which was considered sufficient to evaluate diagnostic concordance between NPS and BALF for common respiratory pathogens. However, the sample size may limit the statistical power for subgroup analyses involving less frequently detected pathogens.

### Two-phase specimen collection

A standardized two-phase specimen collection framework was implemented to evaluate both cross-sectional concordance and longitudinal pathogen dynamics between nasopharyngeal swabs (NPS) and bronchoalveolar lavage fluid (BALF). During the initial diagnostic phase at ICU admission, NPS specimens were collected within a predefined window of ≤2 h prior to the first bronchoalveolar lavage to minimize temporal variability in pathogen detection. Subsequent BALF samples were obtained as part of routine clinical care in accordance with institutional standard operating procedures. The second sampling phase was conducted after a minimum interval of seven days of antimicrobial treatment. Repeat paired NPS and BALF samples were collected only in patients for whom a second diagnostic bronchoscopy was clinically indicated, such as those with persistent or worsening respiratory symptoms, inadequate clinical response to therapy, or suspicion of secondary infection. This approach ensured that all repeat BAL procedures were clinically justified and not driven solely by research considerations. By aligning upper and lower respiratory tract sampling within defined temporal windows and restricting repeat sampling to clinically indicated cases, this two-phase framework was designed to reduce bias related to disease progression, antimicrobial exposure, and procedural timing. The longitudinal component enabled assessment of the ability of NPS-based molecular diagnostics to reflect changes in pathogen profiles over time in comparison with BALF, thereby providing insight into its potential utility for dynamic monitoring in critically ill patients.

### Targeted next-generation sequencing workflow and quality control

Paired specimens underwent tNGS using a clinically validated respiratory panel (Shenzhen Uni-medica Technology Co., Ltd., China) covering 123 bacteria, 37 fungi, 85 viruses and 3 parasites (Supplementary Table S1). The targeted panel includes clinically significant respiratory pathogens frequently associated with antimicrobial resistance in ICU settings, including *Klebsiella pneumoniae, Acinetobacter baumannii*, *Pseudomonas aeruginosa*, and *Staphylococcus aureus*. However, the panel was primarily designed for pathogen identification rather than comprehensive antimicrobial resistance gene profiling. Briefly, 1 mL of specimens were first homogenized with dithiothreitol and then added to a tube containing zirconia beads and lysis buffer. Microbial cells were disrupted by vortexing for 10 min. Nucleic acid was extracted and libraries were then prepared following the manufacturer’s guidelines. Briefly, pathogen DNA and cDNA (generated from RNA by reverse transcription PCR) served as the template for target enrichment. This enrichment was achieved through multiplex PCR amplification utilizing pathogen-specific primers and sample-specific indices within the proprietary “All-In-One” three-primer system. The resulting amplicons were purified twice using magnetic beads with 80% ethanol washes, followed by elution. Subsequently, a second indexing PCR was performed to introduce batch-specific indices and Illumina-compatible adapters. The final indexed libraries underwent another bead purification step, and then were quantified using Qubit 4 Fluorometer (Thermo Fisher Scientific, US) prior to pooling and sequencing. To control for potential reagent and laboratory contamination, a no-template water control was processed concurrently in each run. Sequencing was performed in single-end 50 bp mode on an Illumina MiSeq Dx platform. The bioinformatics analysis pipeline included several steps: quality control of sequencing reads, trimming and filtering of low-quality reads, and alignment against an in-house clinically validated database. Results were reported semi-quantitatively with bins representing approximately <10^3^, 10^4^, 10^5^, or ≥10^6^ genomic copies of nucleic acid per milliliter (copies/mL) of specimen.

### Statistical analysis

Descriptive statistics for continuous variables are reported as median with interquartile range (IQR). Fisher’s exact test was used for analyzing categorical data using contingency tables. A two-tailed *p* value ≤0.05 was considered statistically significant. The PPA, NPA, and positive and negative predictive values (PPV and NPV) of the NPS results were calculated using the paired BALF results as a reference. Statistical analyses were performed with IBM SPSS Statistics (version 25.0). And corresponding 95% confidence intervals (CI) calculated using the Wilson method.

## Results

### Cohort characteristics

A total of 105 critically ill adult patients with severe pneumonia admitted to the intensive care unit were enrolled in the study. The median age of the cohort was 77 years (interquartile range [IQR], 67–87 years), and n = 68 patients (65.0%) were male. Baseline demographic and clinical characteristics are summarized in Supplementary Table S2. All patients fulfilled the ATS/IDSA criteria for severe pneumonia and underwent clinically indicated bronchoalveolar lavage as part of routine diagnostic evaluation. Seventy-four patients (70.5%) completed the predefined two-phase sampling protocol and were included in the longitudinal analysis. The interval between the first and second sampling was a median of 7 days (mean = 6.78 days; range 1–8 days). The remaining 31 patients underwent single-time-point sampling only, primarily due to clinical improvement, resolution of infection, or lack of indication for repeat bronchoscopy. At the time of initial sampling, the majority of patients required advanced respiratory support, including invasive mechanical ventilation or high-flow oxygen therapy, reflecting the severity of illness in the study population. Common underlying comorbidities included chronic cardiovascular disease, chronic pulmonary disease, diabetes mellitus, and cerebrovascular disease. Most patients had received empirical antimicrobial therapy prior to ICU admission or specimen collection, in accordance with standard clinical practice for severe pneumonia. Patients who underwent two-phase sampling were broadly comparable to those with single-phase sampling with respect to age, sex distribution, baseline severity indices, and major comorbidities (Supplementary Table S2), supporting the representativeness of the longitudinal subgroup. No procedure-related serious adverse events were observed during bronchoalveolar lavage in either sampling phase.

### Baseline pathogen profiling

**Figure 1:**
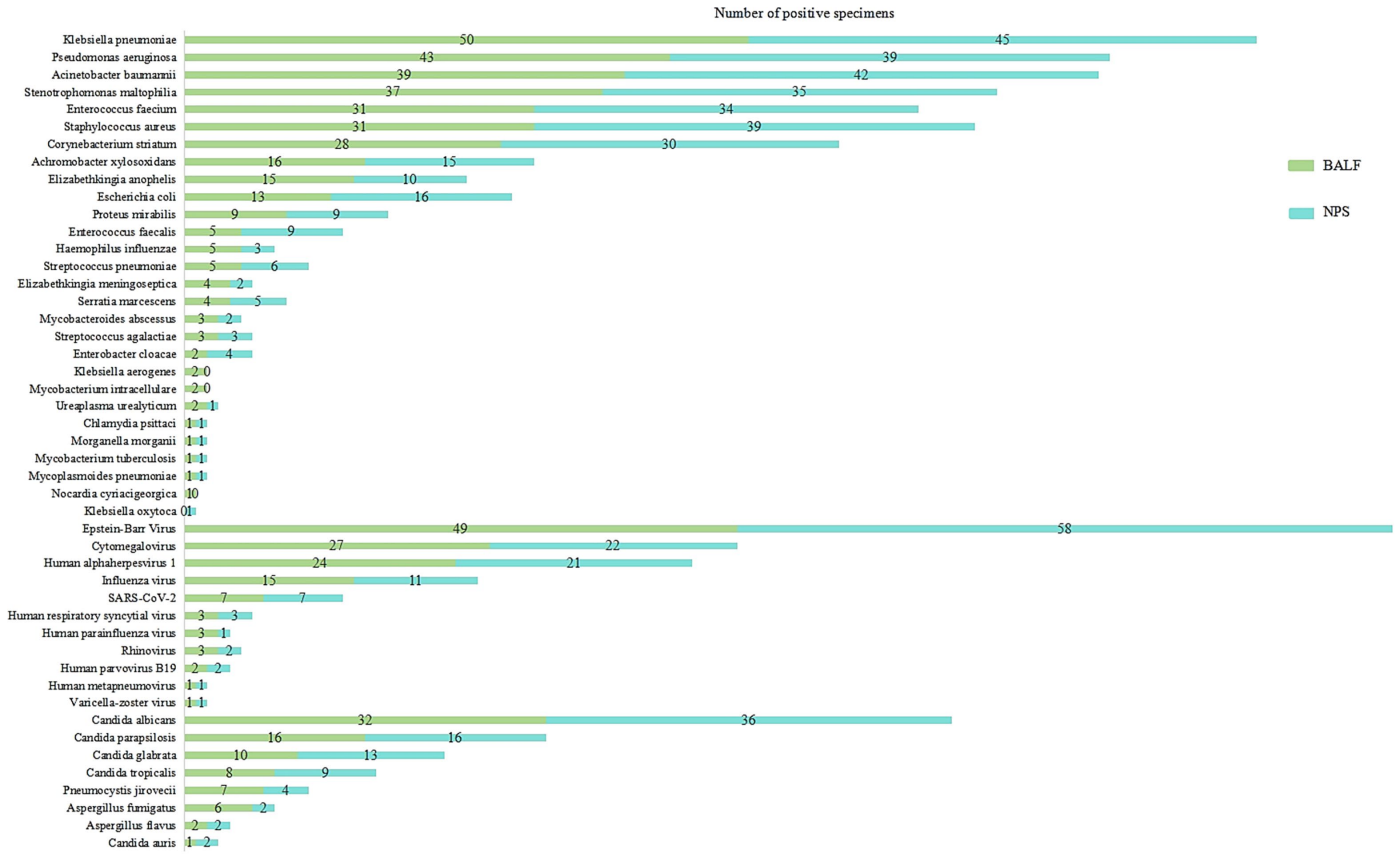
Baseline pathogen profiling identified in 210 paired specimens (105 NPS-BALF pairs).

Baseline targeted next-generation sequencing (tNGS) analysis was performed on 210 paired respiratory specimens, comprising 105 nasopharyngeal swab (NPS) and bronchoalveolar lavage fluid (BALF) pairs. Overall, tNGS identified a broad spectrum of respiratory pathogens, including 28 bacterial species, 11 viral species, and 8 fungal species across the cohort (Figure 1). Bacterial pathogens predominated in both specimen types. *Klebsiella pneumoniae* was the most frequently detected organism, identified in 50 BALF samples and 45 corresponding NPS samples, followed by *Pseudomonas aeruginosa* (n = 43 BALF / n = 39 NPS) and *Acinetobacter baumannii* (n = 39 BALF / n = 35 NPS). These organisms accounted for the majority of bacterial detections in both upper and lower respiratory tract specimens. Additional commonly detected bacteria included *Stenotrophomonas maltophilia*, *Enterococcus faecium*, *Staphylococcus aureus*, *Corynebacterium striatum*, *Achromobacter xylosoxidans*, *Elizabethkingia anophelis*, and *Escherichia coli*, each identified in both BALF and NPS at lower but comparable frequencies. Mycobacterial and atypical bacterial pathogens were detected less frequently. Six mycobacterial isolates were identified in BALF and three in NPS, including *Mycobacteroides abscessus*, *Mycobacterium intracellulare*, and *Mycobacterium tuberculosis*. Similarly, four atypical bacterial detections were observed in BALF and three in NPS, comprising *Ureaplasma urealyticum*, *Chlamydia psittaci*, and *Mycoplasmoides pneumoniae.* Notably, *Klebsiella aerogenes*, *Mycobacterium intracellulare*, and *Nocardia cyriacigeorgica* were detected exclusively in BALF, each in a small number of cases (two, two, and one samples, respectively). Viral pathogens were commonly identified in both specimen types. *Epstein–Barr virus* was the most frequently detected virus (n = 49 BALF / n = 58 NPS), followed by *cytomegalovirus* (n = 27 BALF / n = 22 NPS) and *Human alphaherpesvirus* (n = 24 BALF /n = 21 NPS). *Influenza virus* (n = 15 BALF /n = 11 NPS) and *Severe acute respiratory syndrome coronavirus* 2 (SARS-CoV-2) (n = 7 BALF / n = 7 NPS) were also detected. Other respiratory viruses, including *Human respiratory syncytial virus*, *Human parainfluenza virus*, *Rhinovirus*, and *Human metapneumovirus*, were detected infrequently in both BALF and NPS. Fungal pathogens were predominantly represented by Candida species. *Candida albicans* was detected in n = 32 BALF samples and n = 36 NPS samples, followed by *Candida parapsilosis* (n = 16 BALF / n = 16 NPS) and *Candida glabrata* (n = 10 BALF /n = 13 NPS). *Pneumocystis jirovecii* was identified in seven BALF samples and four NPS samples. Aspergillus species (*Aspergillus fumigatus* and *Aspergillus flavus*) were detected in eight BALF samples and four NPS samples.

### Agreement of pathogen detection between paired NPS and BALF

Agreement of pathogen detection between nasopharyngeal swabs (NPS) and bronchoalveolar lavage fluid (BALF) was evaluated using a total of 179 paired specimens, comprising 105 pairs from the initial sampling and 74 pairs from the second sampling phase (Supplementary Table S2). Agreement metrics for common respiratory pathogens are summarized in Table 1, and a complete overview for all detected pathogens is provided in Supplementary Table S3. BALF was used as the reference standard for calculation of positive percent agreement (PPA), negative percent agreement (NPA), positive predictive value (PPV), and negative predictive value (NPV). For common bacterial pathogens, high concordance between NPS and BALF was observed. The PPA for *Acinetobacter baumannii* was 92.3% (36/39), for *Staphylococcus aureus* 92.3% (24/26), and for *Escherichia coli* 90.0% (18/20). Similarly, PPA was high for *Klebsiella pneumoniae* at 88.5% (46/52), *Pseudomonas aeruginosa* at 88.1% (37/42), and *Streptococcus pneumoniae* at 85.7% (12/14). In contrast, lower PPA values were observed for *Haemophilus influenzae* at 42.9% (3/7) and *Enterobacter cloacae* at 33.3% (1/3). For mycobacterial pathogens, complete agreement was observed for *Mycobacterium tuberculosis* (100.0%, 3/3), while *Mycobacteroides abscessus* demonstrated a PPA of 80.0% (4/5). *Mycobacterium intracellulare* was detected exclusively in BALF and not identified in paired NPS samples.

**Table 1:** Agreement of common pathogen detection between paired NPS and BALF.

Pathogen	NPS+/BALF+	NPS−/BALF+	NPS+/BALF-	NPS−/BALF-	PPA (%) (95% CI)	NPA (%) (95% CI)	PPV (%) (95% CI)	NPV (%) (95% CI)
*Klebsiella pneumoniae*	77	10	10	82	88.5 (80.1–93.6)	89.1 (81.1–94.0)	88.5 (80.1–93.6)	89.1 (81.1–94.0)
*Pseudomonas aeruginosa*	74	10	2	93	88.1 (79.5–93.4)	97.9 (92.6–99.4)	97.4 (90.9–99.3)	90.3 (83.0–94.6)
*Stenotrophomonas maltophilia*	68	13	3	95	83.9 (74.5–90.4)	96.9 (91.4–99.0)	95.8 (88.3–98.6)	88.0 (80.5–92.8)
*Acinetobacter baumannii*	60	5	12	102	92.3 (81.8–97.0)	89.5 (82.5–93.9)	83.3 (73.1–90.2)	95.3 (89.5–98.0)
*Staphylococcus aureus*	48	4	20	107	92.3 (81.8–97.0)	84.2 (76.9–89.6)	70.6 (58.9–80.1)	96.4 (91.1–98.6)
*Escherichia coli*	18	2	11	148	90.0 (69.9–97.2)	93.1 (88.0–96.1)	62.1 (44.0–77.3)	98.7 (95.3–99.6)
*Proteus mirabilis*	10	2	6	161	83.3 (55.2–95.3)	96.4 (92.4–98.3)	62.5 (38.6–81.5)	98.8 (95.8–99.7)
*Serratia marcescens*	6	2	2	169	75.0 (40.9–92.9)	98.8 (95.8–99.7)	75.0 (40.9–92.9)	98.8 (95.8–99.7)
*Streptococcus pneumoniae*	6	1	1	171	85.7 (48.7–97.4)	99.4 (96.8–99.9)	85.7 (48.7–97.4)	99.4 (96.8–99.9)
*Haemophilus influenzae*	3	4	0	172	42.9 (15.8–75.0)	100.0 (97.8–100.0)	100.0 (43.9–100.0)	97.7 (94.3–99.1)
*Enterobacter cloacae*	1	2	4	172	33.3 (6.1–79.2)	97.7 (94.3–99.1)	20.0 (3.6–62.4)	98.8 (95.9–99.7)
*Mycobacteroides abscessus*	4	1	0	174	80.0 (37.6–96.4)	100.0 (97.8–100)	100.0 (51.0–100.0)	99.4 (96.8–99.9)
Influenza virus	17	7	1	154	70.8 (50.8–85.1)	99.3 (96.4–99.9)	94.4 (74.2–99.0)	95.6 (91.3–97.9)
SARS-CoV-2	11	0	0	168	100.0 (74.1–100.0)	100.0 (97.8–100.0)	100.0 (74.1–100.0)	100.0 (97.8–100.0)
Human respiratory syncytial virus	4	1	0	174	80.0 (37.6–96.4)	100.0 (97.8–100.0)	100.0 (51.0–100.0)	99.4 (96.8–99.9)
Human parainfluenza virus	2	3	0	174	40.0 (11.8–76.9)	100.0 (97.8–100.0)	100.0 (34.2–100.0)	98.3 (95.1–99.4)
*Aspergillus fumigatus*	2	8	1	168	20.0 (5.7–51.0)	99.4 (96.7–99.9)	66.7 (66.7–93.9)	95.4 (91.3–97.7)
*Pneumocystis jirovecii*	4	4	0	171	50.0 (21.5–78.5)	100.0 (97.8–100.0)	100.0 (51.0–100.0)	97.7 (94.3–99.1)
*Aspergillus flavus*	0	2	3	174	0.0 (0.0–65.8)	98.3 (95.1–99.4)	0.0 (0.0–56.1)	98.9 (96.0–99.7)

NPS, Nasopharyngeal swab; BALF, Bronchoalveolar lavage fluid; PPA, Positive percent agreement; NPA, Negative percent agreement; PPV, Positive predictive value; NPV, Negative predictive value.

For viral pathogens, NPS showed moderate agreement with BALF. PPA values were ≤80.0% for most viruses, whereas complete agreement was observed for severe acute respiratory syndrome coronavirus 2 (SARS-CoV-2) (100.0%, 7/7). Fungal pathogen detection demonstrated substantial discordance between specimen types. *Aspergillus* species were predominantly detected in BALF, with PPA of 20.0% (1/5) for *Aspergillus fumigatus* and 0.0% (0/3) for *Aspergillus flavus*. *Pneumocystis jirovecii* was detected in four paired NPS–BALF samples and in four BALF-only samples, corresponding to a PPA of 50.0% (4/8).

Across pathogens, PPV was ≥80.0% for most organisms and reached ≥95.0% for *Pseudomonas aeruginosa*, *Stenotrophomonas maltophilia*, *Haemophilus influenzae*, *Mycobacteroides abscessus*, *Pneumocystis jirovecii*, and most viral pathogens. Lower PPV values were observed for *Staphylococcus aureus* (70.6%, 24/34), *Escherichia coli* (62.1%, 18/29), *Proteus mirabilis* (62.5%, 5/8), *Serratia marcescens* (75.0%, 6/8), *Enterobacter cloacae* (20.0%, 1/5), and *Aspergillus* species (0.0–66.7%). NPA and NPV were ≥95.0% for most pathogens but were lower for frequently detected bacteria including *Klebsiella pneumoniae*, *Pseudomonas aeruginosa*, *Stenotrophomonas maltophilia*, *Acinetobacter baumannii*, *Staphylococcus aureus*, and *Escherichia coli*. The commonly organisms detected but infrequently implicated as primary respiratory pathogens—such as *Enterococcus faecium*, *Corynebacterium striatum*, *Epstein–Barr virus*, *Cytomegalovirus*, *Human alphaherpesvirus*, and *Candida* species agreement metrics varied widely. Across these pathogens, PPA ranged from 50.0% (*Candida glabrata*) to 97.9% (*Corynebacterium striatum*), NPA from 77.4% (*Epstein–Barr virus*) to 99.3% (*Elizabethkingia anophelis*), PPV from 50.0% (*Candida auris*) to 95.0% (*Elizabethkingia anophelis*), and NPV from 84.7% (*Epstein–Barr virus*) to 98.8% (*Candida auris*) (Supplementary Table S3).

### Dynamic pathogen monitoring with NPS

To assess the NPS’s capacity for dynamic pathogen monitoring, we selected 328 pathogens that were detected in both type of specimens from 74 patients who completed longitudinal sampling. The semi-quantitative results from the first and second sampling were compared separately for NPS and BALF. A 10-fold or greater difference in relative concentration (copies/mL) was considered a “change.” Agreement between paired NPS and BALF was then examined. For NPS, n = 67 (20.4%) pathogens were not detected in the second sampling (Supplementary Table S2). The relative concentration of 85 (25.9%) pathogens increased and 81 (24.7%) decreased, and no change was observed for 95 (29.1%). The data for BALF was 87 (26.5%), 72 (21.9%), 88 (26.8%) and 81 (24.7%), respectively. The change agreement between NPS and BALF for common pathogens was shown in Table 2. Agreement was higher for *Staphylococcus aureus* (72.7%), *Acinetobacter baumannii* (62.5%) and fungi (*Aspergillus fumigatus* and *Pneumocystis jirovecii*) (75.0%), but poor for *Stenotrophomonas maltophilia* (33.3%) and viruses (14.3–50.0%).

**Table 2:** The change agreement between paired NPS and BALF for common pathogens.

Pathogens	Change agreement	
	Yes	No
*Pseudomonas aeruginosa*	18 (54.5%)	15 (45.5%)
*Klebsiella pneumoniae*	17 (53.1%)	15 (46.9%)
*Staphylococcus aureus*	16 (72.7%)	6 (27.3%)
*Acinetobacter baumannii*	15 (62.5%)	9 (37.5%)
*Stenotrophomonas maltophilia*	9 (33.3%)	18 (66.7%)
Influenza virus	4 (50.0%)	4 (50.0%)
Other viruses^*^	1 (14.3%)	6 (85.7%)
Fungi^#^	3 (75.0%)	1 (25.0%)

*Human respiratory syncytial virus, SARS-CoV-2, human parainfluenza virus and rhinovirus. ^#^*Aspergillus fumigatus* and *Pneumocystis jirovecii*.

## Discussion

In this prospective cohort study of critically ill patients with severe pneumonia, we systematically evaluated the agreement of pathogen detection between paired NPS and BALF using targeted next-generation sequencing. By integrating cross-sectional and longitudinal sampling, our findings provide a detailed assessment of the diagnostic performance of upper respiratory tract specimens for bacterial, viral, and fungal pathogens in an ICU population. Our results demonstrated high positive percent agreement (PPA) between NPS and BALF for several clinically important bacterial pathogens commonly associated with severe and nosocomial pneumonia. In particular, *Klebsiella pneumoniae*, *Pseudomonas aeruginosa*, *Stenotrophomonas maltophilia*, *Acinetobacter baumannii*, and *Staphylococcus aureus* showed consistently high PPA values (≥88%), indicating strong concordance between upper and lower respiratory tract specimens for these organisms. These pathogens are well-recognized causes of hospital-acquired and ventilator-associated pneumonia in ICU settings, Many of these organisms, particularly *Klebsiella pneumoniae*, *Acinetobacter baumannii*, and *Pseudomonas aeruginosa*, are also frequently associated with multidrug-resistant phenotypes, further highlighting the clinical importance of rapid molecular detection for guiding early antimicrobial therapy, where bacterial burden is often high and dissemination throughout the respiratory tract may occur (Dandagi, 2010; Webber et al., 2020). These findings contrast with those reported in patients with community-acquired pneumonia (CAP). In a prior study evaluating paired upper and lower respiratory tract specimens in CAP, the PPA ranged from 0% for *Acinetobacter baumannii* to 71.0% for *Pseudomonas aeruginosa* (Serigstad et al., 2023). Similarly, another study reported variable agreement for common bacterial pathogens when comparing NPS with lower respiratory tract samples (Jurado-Martín et al., 2024; Phoo et al., 2025). Differences between these studies and our findings likely reflect variation in patient populations, underlying disease severity, antimicrobial exposure, and pathogen ecology. ICU patients with severe pneumonia may have higher pathogen loads and more extensive airway involvement, potentially increasing the detectability of pathogens in the upper respiratory tract. For *Streptococcus pneumoniae* and *Haemophilus influenzae*, we observed PPA values of 85.7 and 42.9%, respectively. Previous studies have reported a wide range of PPA values for these organisms, from 58.0 to 86.0% (Nair and Niederman, 2021). Direct comparison across studies is challenging due to heterogeneity in specimen types (nasopharyngeal swab, oropharyngeal swab, sputum, and bronchoalveolar lavage fluid), diagnostic platforms (culture-based methods versus molecular assays), and reference standards used for pathogen attribution (Gadsby et al., 2016). In addition, *Streptococcus pneumoniae* and *Haemophilus influenzae* are frequent colonizers of the upper respiratory tract, particularly in elderly individuals and patients with chronic comorbidities, which may inflate concordance metrics while reducing diagnostic specificity for true lower respiratory tract infection (Thevaranjan et al., 2016; Moriki et al., 2025). The consistently high negative percent agreement (NPA) observed for most bacterial pathogens in the present study supports the potential utility of nasopharyngeal swabs in ruling out selected bacterial etiologies in appropriate clinical contexts. However, the lower NPA observed for highly prevalent pathogens such as *Klebsiella pneumoniae* and *Acinetobacter baumannii* underscores the influence of background colonization and pathogen prevalence on agreement metrics, a phenomenon that has been well described in molecular respiratory diagnostics.

Mycobacterial detection showed marked heterogeneity. Complete agreement was observed for *Mycobacterium tuberculosis* (PPA 100%), while *Mycobacteroides abscessus* demonstrated high but incomplete agreement (80%). In contrast, *Mycobacterium intracellulare* was detected exclusively in BALF. The small number of cases precludes definitive interpretation; however, these findings are consistent with previous reports demonstrating variable sensitivity of upper respiratory tract specimens for mycobacterial detection. A systematic review assessing oral swabs for molecular diagnosis of pulmonary tuberculosis reported sensitivities ranging from 36 to 91% in adults and 5 to 42% in children (Church et al., 2024; Moriki et al., 2025). Variability across studies has been attributed to differences in sampling site, swab type, processing methods, and nucleic acid amplification techniques. Our results further underscore the inconsistency of NPS-based detection for non-tuberculous mycobacteria in particular. For respiratory viruses, NPS demonstrated moderate agreement with BALF, with notable pathogen-specific differences. Influenza virus showed a PPA of 70.8%, whereas complete agreement was observed for SARS-CoV-2. These findings are broadly consistent with previous work reporting an overall agreement of approximately 83.7% between NPS and BALF for viral detection in adults with suspected pneumonia (Loubet et al., 2017). Differences between influenza virus and SARS-CoV-2 detection may reflect pathogen-specific replication dynamics and tissue tropism. In critically ill patients, viral loads may differ substantially between the upper and lower respiratory tract. A prior study reported a significant gradient in viral load between NPS and BALF, with lower cycle threshold values in BALF for influenza virus, while such a gradient was less pronounced for SARS-CoV-2 (Chew et al., 2024). In our cohort, similar patterns were observed, suggesting that BALF remains essential for accurate viral detection when upper respiratory tract samples are negative, particularly for influenza virus.

Our study provides novel data on fungal pathogen agreement between NPS and BALF using tNGS. Overall, fungal detection showed poor concordance, particularly for *Aspergillus* species and *Pneumocystis jirovecii*. PPA values were low for *Aspergillus fumigatus* (20%) and absent for *Aspergillus flavus*, while *Pneumocystis jirovecii* demonstrated only moderate agreement (50%). These findings are consistent with known challenges in diagnosing pulmonary fungal infections using upper respiratory tract specimens. Colonization of both upper and lower airways by *Candida* species and environmental molds is common, particularly in critically ill or immunocompromised patients (Ponce et al., 2010; Gago et al., 2019). Moreover, fungal burden may be localized to the lower respiratory tract, limiting detectability in NPS. Our data suggest that NPS has limited utility for diagnosing invasive fungal pneumonia, even when using sensitive molecular platforms such as tNGS.

Across pathogens, PPV and NPV varied substantially and were strongly influenced by pathogen prevalence. High PPV was observed for several bacterial and viral pathogens, while lower PPV for organisms such as *Staphylococcus aureus*, *Escherichia coli*, and *Enterobacter cloacae* highlights the challenge of distinguishing infection from colonization. Importantly, excellent NPA and NPV for most pathogens suggest that negative NPS results may help exclude certain etiologies, although interpretation must remain pathogen-specific. Molecular detection does not inherently distinguish active infection from colonization, asymptomatic carriage, or prolonged shedding following prior infection (Shang et al., 2025). This distinction is particularly important for organisms frequently detected in the upper respiratory tract, including Epstein–Barr virus, *cytomegalovirus*, human a*lphaherpesvirus*, *Candida* species, and commensal bacteria such as *Corynebacterium* spp. In critically ill patients, these organisms may represent colonization, viral reactivation, or bystander detection rather than true etiologic pathogens. Therefore, interpretation of molecular results should always be integrated with clinical findings, radiologic evidence, and host immune status. The longitudinal component of this study revealed limited agreement between NPS and BALF for dynamic pathogen monitoring. Higher agreement was observed only for *Staphylococcus aureus*, *Acinetobacter baumannii*, and selected fungal pathogens. For many organisms, discordance arose from stable detection in one specimen type accompanied by changes in relative abundance in the other. These findings suggest that semi-quantitative tNGS results from upper respiratory tract specimens may not reliably reflect pathogen dynamics in the lower respiratory tract, particularly during antimicrobial treatment.

Importantly, the longitudinal component of this study adds a novel dimension to the evaluation of NPS performance. While cross-sectional agreement was high for several key bacterial pathogens, dynamic changes in pathogen abundance were not consistently mirrored between NPS and BALF over time. Higher concordance was observed only for selected organisms, including *Staphylococcus aureus*, *Acinetobacter baumannii*, and fungal pathogens. These findings emphasize that, although NPS-based tNGS is well suited for initial pathogen identification, BALF remains the most reliable specimen for assessing temporal changes in pathogen burden during the clinical course of severe pneumonia. This study has several limitations. First, the sample size was relatively modest and may limit statistical power for subgroup analyses of less frequently detected pathogens. Second, as bronchoalveolar lavage was performed only when clinically indicated, the study population represents a subset of ICU patients with severe pneumonia and may not fully reflect all cases of lower respiratory tract infection. Third, longitudinal sampling was performed only in patients requiring repeat bronchoscopy, which may introduce additional selection bias. Future multicenter studies with larger cohorts would help validate these findings.

## Conclusion

This prospective cohort study demonstrates that nasopharyngeal swabs analyzed by targeted next-generation sequencing provide high concordance with bronchoalveolar lavage fluid for the detection of major bacterial pathogens responsible for severe pneumonia in ICU patients. NPS-based molecular testing shows excellent negative agreement for most pathogens and strong positive agreement for common nosocomial bacteria, supporting its clinical utility as a less invasive diagnostic approach for initial etiological assessment. However, pathogen-specific differences were observed for viral and fungal infections, and longitudinal analyses indicate limited concordance for dynamic pathogen monitoring. Collectively, these findings highlight the complementary roles of NPS and BALF in molecular diagnostics of severe pneumonia and support the selective integration of noninvasive sampling strategies into ICU diagnostic workflows.

## Data Availability

The data presented in the study are deposited in Genome Sequence Archive repository, accession number CRA041213 (https://ngdc.cncb.ac.cn/gsa/search?searchTerm=CRA041213).

## References

[ref1] Cao B. Huang Y. She D.-Y. Cheng Q.-J. Fan H. Tian X.-L. . (2018). Diagnosis and treatment of community-acquired pneumonia in adults: 2016 clinical practice guidelines by the Chinese thoracic society, Chinese Medical Association. Clin. Respir. J. 12, 1320–1360. doi: 10.1111/crj.12674, 28756639 PMC7162259

[ref2] Chew R. Tozer S. Ulett K. Paterson D. L. Whiley D. Sloots T. . (2024). Comparing polymerase chain reaction testing of nasopharyngeal swab and lower respiratory tract specimens for the diagnosis of *pneumocystis jirovecii* pneumonia. Open Forum Infect. Dis. 11:ofae071. doi: 10.1093/ofid/ofae071, 38444816 PMC10913836

[ref3] Church E. C. Steingart K. R. Cangelosi G. A. Ruhwald M. Kohli M. Shapiro A. E. (2024). Oral swabs with a rapid molecular diagnostic test for pulmonary tuberculosis in adults and children: a systematic review. Lancet Glob. Health 12, e45–e54. doi: 10.1016/S2214-109X(23)00469-2, 38097297 PMC10733129

[ref4] Dandagi G. L. (2010). Nosocomial pneumonia in critically ill patients. Lung India 27, 149–153. doi: 10.4103/0970-2113.68321, 20931034 PMC2946717

[ref5] Demars Y. Brahier T. Rotzinger D. C. Brouillet R. Jaton K. Opota O. . (2022). Utility of polymerase chain reaction in nasopharyngeal swabs for identifying respiratory Bacteria causing community-acquired pneumonia. Microbiol. Spectrum 10:e0037922. doi: 10.1128/spectrum.00379-22, 35583335 PMC9241648

[ref6] Gadsby N. J. Russell C. D. McHugh M. P. Mark H. Conway Morris A. Laurenson I. F. . (2016). Comprehensive molecular testing for respiratory pathogens in community-acquired pneumonia. Clin. Infect. Dis. 62, 817–823. doi: 10.1093/cid/civ1214, 26747825 PMC4787606

[ref7] Gago S. Denning D. W. Bowyer P. (2019). Pathophysiological aspects of *aspergillus* colonization in disease. Med. Mycol. 57:S219–S227. doi: 10.1093/mmy/myy076, 30239804

[ref8] Hattab S. Ma A. H. Tariq Z. Prado I. V. Drobish I. Lee R. . (2024). Rapid phenotypic and genotypic antimicrobial susceptibility testing approaches for use in the clinical laboratory. Antibiotics 13:786. doi: 10.3390/antibiotics13080786, 39200086 PMC11351821

[ref9] Jurado-Martín I. Ma C. Rezk N. Sainz-Mejías M. Hou Y. Baugh J. A. . (2024). Development of acute *Pseudomonas aeruginosa* and *Acinetobacter baumannii* lung mono-challenge models in mice using oropharyngeal aspiration. Access Microbiol. 6:000860.v3. doi: 10.1099/acmi.0.000860.v3, 39575441 PMC11580749

[ref10] Kamel T. Helms J. Janssen-Langenstein R. Kouatchet A. Guillon A. Bourenne J. . (2020). Benefit-to-risk balance of bronchoalveolar lavage in the critically ill. A prospective, multicenter cohort study. Intensive Care Med. 46, 463–474. doi: 10.1007/s00134-019-05896-4, 31912201 PMC7223716

[ref11] Loubet P. Voiriot G. Houhou-Fidouh N. Neuville M. Bouadma L. Lescure F.-X. . (2017). Impact of respiratory viruses in hospital-acquired pneumonia in the intensive care unit: a single-center retrospective study. J. Clin. Virol. 91, 52–57. doi: 10.1016/j.jcv.2017.04.001, 28494435 PMC7106511

[ref12] Messacar K. Parker S. K. Todd J. K. Dominguez S. R. (2017). Implementation of rapid molecular infectious disease diagnostics: the role of diagnostic and antimicrobial stewardship. J. Clin. Microbiol. 55, 715–723. doi: 10.1128/JCM.02264-16, 28031432 PMC5328439

[ref13] Metlay J. P. Waterer G. W. Long A. C. Anzueto A. Brozek J. Crothers K. . (2019). Diagnosis and treatment of adults with community-acquired pneumonia. An official clinical practice guideline of the American Thoracic Society and Infectious Diseases Society of America. Am. J. Respir. Crit. Care Med. 200, e45–e67. doi: 10.1164/rccm.201908-1581ST, 31573350 PMC6812437

[ref14] Moriki D. Tsouprou M. Prountzos S. Koumpagioti D. Kalogiannis M. Alexopoulou E. . (2025). Bacterial isolates from Bronchoalveolar lavage in pediatric patients with protracted bacterial bronchitis or bronchiectasis: a retrospective comparative study. J. Clin. Med. 14:7653. doi: 10.3390/jcm14217653, 41227049 PMC12610843

[ref15] Murdoch D. R. (2016). How recent advances in molecular tests could impact the diagnosis of pneumonia. Expert. Rev. Mol. Diagn. 16, 533–540. doi: 10.1586/14737159.2016.1156536, 26891612 PMC7103682

[ref16] Nair G. B. Niederman M. S. (2021). Updates on community acquired pneumonia management in the ICU. Pharmacol. Ther. 217:107663. doi: 10.1016/j.pharmthera.2020.107663, 32805298 PMC7428725

[ref17] Nyawanda B. O. Njuguna H. N. Onyango C. O. Makokha C. Lidechi S. Fields B. . (2019). Comparison of respiratory pathogen yields from nasopharyngeal/oropharyngeal swabs and sputum specimens collected from hospitalized adults in rural Western Kenya. Sci. Rep. 9:11237. doi: 10.1038/s41598-019-47713-4, 31375774 PMC6677726

[ref18] Phoo M. T. P. Dechathai T. Singkhamanan K. Chusri S. Pomwised R. Wonglapsuwan M. . (2025). *Pseudomonas aeruginosa* affects *Acinetobacter baumannii*’s growth, gene expression and antibiotic resistance in in vitro co-culture system. Curr Res Microb Sci 9:100499. doi: 10.1016/j.crmicr.2025.100499, 41246283 PMC12613057

[ref19] Ponce C. A. Gallo M. Bustamante R. Vargas S. L. (2010). Pneumocystis colonization is highly prevalent in the autopsied lungs of the general population. Clin. Infect. Dis. 50, 347–353. doi: 10.1086/649868, 20047487

[ref20] Serigstad S. Knoop S. T. Markussen D. L. Ulvestad E. Bjørneklett R. O. Ebbesen M. H. . (2023). Diagnostic utility of oropharyngeal swabs as an alternative to lower respiratory tract samples for PCR-based syndromic testing in patients with community-acquired pneumonia. J. Clin. Microbiol. 61:e0050523. doi: 10.1128/jcm.00505-23, 37585220 PMC10512787

[ref21] Shang H. Zou S. Ma Z. Liang Q. Zhong Y. Li L. . (2025). Comparative and clinical impact of targeted next-generation sequencing in pediatric pneumonia diagnosis and treatment. Front. Microbiol. 16:1590792. doi: 10.3389/fmicb.2025.1590792, 40636506 PMC12238002

[ref22] Thevaranjan N. Whelan F. J. Puchta A. Ashu E. Rossi L. Surette M. G. . (2016). *Streptococcus pneumoniae* colonization disrupts the microbial community within the upper respiratory tract of aging mice. Infect. Immun. 84, 906–916. doi: 10.1128/IAI.01275-15, 26787714 PMC4807479

[ref23] Torres A. Fernández-Barat L. (2014). New developments in the diagnosis of VAP make bronchoalveolar lavage less useful: some considerations. Intensive Care Med. 40, 1778–1779. doi: 10.1007/s00134-014-3466-6, 25209130

[ref24] Torres A. Niederman M. S. Chastre J. Ewig S. Fernandez-Vandellos P. Hanberger H. . (2017). International ERS/ESICM/ESCMID/ALAT guidelines for the management of hospital-acquired pneumonia and ventilator-associated pneumonia: guidelines for the management of hospital-acquired pneumonia (HAP)/ventilator-associated pneumonia (VAP) of the European Respiratory Society (ERS), European Society of Intensive Care Medicine (ESICM), European Society of Clinical Microbiology and Infectious Diseases (ESCMID) and Asociación Latinoamericana del Tórax (ALAT). Eur. Respir. J. 50:1700582. doi: 10.1183/13993003.00582-2017, 28890434

[ref25] Webber D. M. Wallace M. A. Burnham C.-A. D. Anderson N. W. (2020). Evaluation of the BioFire FilmArray pneumonia panel for detection of viral and bacterial pathogens in lower respiratory tract specimens in the setting of a tertiary care Academic Medical Center. J. Clin. Microbiol. 58, e00343–e00320. doi: 10.1128/JCM.00343-20, 32321782 PMC7315030

[ref26] Zhang P. Liu B. Zhang S. Chang X. Zhang L. Gu D. . (2024). Clinical application of tasrgeted next-generation sequencing in severe pneumonia: a retrospective review. Crit. Care 28:225. doi: 10.1186/s13054-024-05009-8, 38978111 PMC11232260

