# Supplementary material for: Non-invasive molecular surveillance of drug-resistant bacterial and fungal pathogens in severe ICU pneumonia: a comparative study of nasopharyngeal swabs and BALF
Source: Front Microbiol. 2026 Apr 22;17:1794266. doi: 10.3389/fmicb.2026.1794266 (PMC13143981; doi:10.3389/fmicb.2026.1794266)
Supplement: Supplementary file 1 [file Table_1.docx]

**Supplementary Material**

| **Table S1.** Pathogens include in the tNGS panel. | |
| --- | --- |
| **Category** | **Pathogen** |
| Bacteria (123) | *Staphylococcus aureus* |
|  | *Staphylococcus epidermidis* |
|  | *Staphylococcus haemolyticus* |
|  | *Staphylococcus hominis* |
|  | *Streptococcus pneumoniae* |
|  | *Streptococcus pyogenes* |
|  | *Streptococcus dysgalactiae* |
|  | *Streptococcus intermedius* |
|  | *Streptococcus mitis* |
|  | *Streptococcus suis* |
|  | *Streptococcus constellatus* |
|  | *Streptococcus agalactiae* |
|  | *Streptococcus anginosus* |
|  | *Enterococcus faecium* |
|  | *Enterococcus faecalis* |
|  | *Enterococcus casseliflavus* |
|  | *Nocardia* |
|  | *Nocardia farcinica* |
|  | *Nocardia cyriacigeorgica* |
|  | *Nocardia brasiliensis* |
|  | *Nocardia concava* |
|  | *Nocardia asteroides* |
|  | *Mycobacterium tuberculosis* |
|  | *Mycobacterium avium* |
|  | *Mycobacterium intracellulare* |
|  | *Mycobacteroides abscessus* |
|  | *Mycobacterium kansasii* |
|  | *Mycobacteroides chelonae* |
|  | *Mycolicibacterium fortuitum* |
|  | *Mycobacterium marinum* |
|  | *Mycobacterium haemophilum* |
|  | *Mycobacterium gordonae* |
|  | *Mycobacterium xenopi* |
|  | *Mycolicibacterium vaccae* |
|  | *Mycobacterium malmoense* |
|  | *Mycobacterium scrofulaceum* |
|  | *Mycobacterium ulcerans* |
|  | *Mycobacterium simiae* |
|  | *Mycolicibacterium diernhoferi* |
|  | *Mycobacterium lepromatosis* |
|  | *Mycobacterium parascrofulaceum* |
|  | *Mycobacterium szulgai* |
|  | *Mycolicibacillus trivialis* |
|  | *Mycolicibacter nonchromogenicus* |
|  | *Mycolicibacter terrae* |
|  | *Mycolicibacterium gilvum* |
|  | *Mycolicibacterium neoaurum* |
|  | *Mycolicibacterium phlei* |
|  | *Mycolicibacterium smegmatis* |
|  | *Bacillus anthracis* |
|  | *Bacillus cereus group* |
|  | *Gordonia bronchialis* |
|  | *Rhodococcus equi* |
|  | *Trueperella pyogenes* |
|  | *Tropheryma whipplei* |
|  | *Actinomyces israelii* |
|  | *Actinomyces naeslundii* |
|  | *Parvimonas micra* |
|  | *Corynebacterium diphtheriae* |
|  | *Corynebacterium pseudotuberculosis* |
|  | *Corynebacterium striatum* |
|  | *Listeria monocytogenes* |
|  | *Klebsiella pneumoniae* |
|  | *Klebsiella oxytoca* |
|  | *Klebsiella aerogenes* |
|  | *Pseudomonas aeruginosa* |
|  | *Stenotrophomonas maltophilia* |
|  | *Escherichia coli* |
|  | *Moraxella catarrhalis* |
|  | *Enterobacter cloacae* |
|  | *Serratia marcescens* |
|  | *Burkholderia cepacia* |
|  | *Proteus mirabilis* |
|  | *Acinetobacter* |
|  | *Acinetobacter baumannii* |
|  | *Haemophilus influenzae* |
|  | *Haemophilus parainfluenzae* |
|  | *Haemophilus haemolyticus* |
|  | *Bordetella pertussis* |
|  | *Bordetella parapertussis* |
|  | *Bordetella holmesii* |
|  | *Bordetella avium* |
|  | *Bordetella bronchiseptica* |
|  | *Morganella morganii* |
|  | *Legionella pneumophila* |
|  | *Legionella longbeachae* |
|  | *Achromobacter denitrificans* |
|  | *Raoultella ornithinolytica* |
|  | *Raoultella planticola* |
|  | *Achromobacter xylosoxidans* |
|  | *Bacteroides fragilis* |
|  | *Aeromonas hydrophila* |
|  | *Brucella* |
|  | *Sphingomonas paucimobilis* |
|  | *Elizabethkingia anophelis* |
|  | *Neisseria meningitidis* |
|  | *Burkholderia mallei* |
|  | *Burkholderia pseudomallei* |
|  | *Elizabethkingia meningoseptica* |
|  | *Yersinia pestis* |
|  | *Salmonella enterica subsp. enterica* |
|  | *Francisella tularensis* |
|  | *Pasteurella multocida* |
|  | *Fusobacterium necrophorum* |
|  | *Chryseobacterium indologenes* |
|  | *Chromobacterium violaceum* |
|  | *Mycoplasmoides pneumoniae* |
|  | *Chlamydia pneumoniae* |
|  | *Chlamydia psittaci* |
|  | *Chlamydia trachomatis* |
|  | *Ureaplasma urealyticum* |
|  | *Ureaplasma parvum* |
|  | *Mycoplasmoides genitalium* |
|  | *Leptospira interrogans* |
|  | *Bartonella* |
|  | *Orientia tsutsugamushi* |
|  | *Rickettsia typhi* |
|  | *Rickettsia prowazekii* |
|  | *Rickettsia rickettsii* |
|  | *Rickettsia conorii* |
|  | *Rickettsia felis* |
|  | *Coxiella burnetii* |
|  | *Anaplasma phagocytophilum* |
| Fungus (37) | *Aspergillus* |
|  | *Aspergillus fumigatus* |
|  | *Aspergillus flavus* |
|  | *Aspergillus terreus* |
|  | *Aspergillus niger* |
|  | *Aspergillus nidulans* |
|  | *Aspergillus oryzae* |
|  | *Candida albicans* |
|  | *Candida parapsilosis* |
|  | *Candida tropicalis* |
|  | *Nakaseomyces glabratus* |
|  | *Candida auris* |
|  | *Pneumocystis jirovecii* |
|  | *Cryptococcus neoformans species complex* |
|  | *Cryptococcus gattii species complex* |
|  | *Talaromyces marneffei* |
|  | *Histoplasma capsulatum* |
|  | *Sporothrix schenckii* |
|  | *Blastomyces dermatitidi* |
|  | *Coccidioides immitis* |
|  | *Scedosporium apiospermum* |
|  | *Scedosporium* |
|  | *Lomentospora prolificans* |
|  | *Mucor* |
|  | *Mucor racemosus* |
|  | *Rhizopus* |
|  | *Rhizopus microsporus* |
|  | *Rhizopus oryzae* |
|  | *Rhizomucor* |
|  | *Rhizomucor pusillus* |
|  | *Lichtheimia* |
|  | *Lichtheimia corymbifera* |
|  | *Cunninghamella* |
|  | *Cunninghamella bertholletiae* |
|  | *Schizophyllum commune* |
|  | *Trichosporon asahii* |
|  | *Fusarium* |
| Virus (85) | Human mastadenovirus B |
|  | Human adenovirus B3 |
|  | Human adenovirus 7 |
|  | Human adenovirus 55 |
|  | Human mastadenovirus C |
|  | Human adenovirus 1 |
|  | Human adenovirus 2 |
|  | Human adenovirus 5 |
|  | Human adenovirus 6 |
|  | Human mastadenovirus E |
|  | Human adenovirus E4 |
|  | Human bocavirus 1 |
|  | Human bocavirus 2 |
|  | Human bocavirus 3 |
|  | Human bocavirus 4 |
|  | Human alphaherpesvirus 1 |
|  | Human alphaherpesvirus 2 |
|  | Varicella-zoster virus |
|  | Epstein-Barr Virus |
|  | Cytomegalovirus |
|  | Human betaherpesvirus 6 |
|  | Human betaherpesvirus 7 |
|  | BK Polyomavirus |
|  | JC polyomavirus |
|  | KI Polyomavirus |
|  | WU Polyomavirus |
|  | Human parvovirus B19 |
|  | Enterovirus |
|  | Enterovirus A |
|  | Enterovirus A71 |
|  | Coxsackievirus A2 |
|  | Coxsackievirus A6 |
|  | Coxsackievirus A10 |
|  | Coxsackievirus A16 |
|  | Enterovirus B |
|  | Coxsackievirus A9 |
|  | Coxsackievirus B1 |
|  | Coxsackievirus B2 |
|  | Coxsackievirus B3 |
|  | Coxsackievirus B5 |
|  | Coxsackievirus B6 |
|  | Echovirus E11 |
|  | Echovirus E30 |
|  | Enterovirus C |
|  | Enterovirus D |
|  | Enterovirus D68 |
|  | Rhinovirus A |
|  | Rhinovirus B |
|  | Rhinovirus C |
|  | Human respiratory syncytial virus A |
|  | Human respiratory syncytial virus B |
|  | Influenza A virus |
|  | Influenza A virus H1N1 |
|  | Influenza A virus H3N2 |
|  | Influenza A virus H5N1 |
|  | Influenza A virus H7N9 |
|  | Influenza B virus |
|  | Influenza C virus |
|  | Human respirovirus 1 |
|  | Human orthorubulavirus 2 |
|  | Human respirovirus 3 |
|  | Human orthorubulavirus 4 |
|  | Human metapneumovirus |
|  | Measles virus |
|  | Rubella virus |
|  | Mumps virus |
|  | Hendra virus |
|  | Nipah virus |
|  | Severe acute respiratory syndrome coronavirus |
|  | Middle East respiratory syndrome-related coronavirus |
|  | Human coronavirus 229E |
|  | Human coronavirus HKU1 |
|  | Human coronavirus NL63 |
|  | Human coronavirus OC43 |
|  | Severe acute respiratory syndrome coronavirus 2 |
|  | Severe acute respiratory syndrome coronavirus 2 （Omicron variant) |
|  | Hantavirus |
|  | Seoul virus |
|  | Dengue virus |
|  | Dengue virus type 1 |
|  | Dengue virus type 2 |
|  | Dengue virus type 3 |
|  | Dengue virus type 4 |
|  | Parechovirus A |
|  | Rotavirus |
| Parasite (3) | *Paragonimus westermani* |
|  | *Entamoeba histolytica* |
|  | *Cryptosporidium* |

| **Table S2. Patient characterises and pathogen findings in NPS and BALF by tNGS.** | | | | | | | | | | | | | | |
| --- | --- | --- | --- | --- | --- | --- | --- | --- | --- | --- | --- | --- | --- | --- |
| **Patient ID** | **Age(years)** | **Gender** | **Pathogens** | **First sampling (copies/mL)** | | |  | **Second sampling (copies/mL)** | | |  | **Change between two sampling** | | **Change agreement** |
|  |  |  |  | **NPS** | **BALF** | **Consistent** |  | **NPS** | **BALF** | **Consistent** |  | **NPS** | **BALF** |  |
| P1 | 44 | Male | Chlamydia psittaci | ≥10^6 | ≥10^6 | Yes |  | - | - | - |  | - | - | - |
|  |  |  | Influenza virus | 10^5 | 10^5 | Yes |  | - | - | - |  | - | - | - |
|  |  |  | Streptococcus pneumoniae | 10^3 | Not detected | No |  | - | - | - |  | - | - | - |
|  |  |  | Staphylococcus aureus | <10^3 | Not detected | No |  | - | - | - |  | - | - | - |
| P2 | 91 | Male | Candida albicans | 10^4 | 10^3 | Yes |  | Not detected | Not detected | Yes |  | Not detected | Not detected | Yes |
|  |  |  | Candida parapsilosis | 10^4 | <10^3 | Yes |  | Not detected | Not detected | Yes |  | Not detected | Not detected | Yes |
|  |  |  | Epstein-Barr virus | 10^4 | <10^3 | Yes |  | <10^3 | <10^3 | Yes |  | Decrease | No change | No |
|  |  |  | Candida glabrata | 10^3 | 10^3 | Yes |  | Not detected | Not detected | Yes |  | Not detected | Not detected | Yes |
|  |  |  | Enterococcus faecium | <10^3 | 10^3 | Yes |  | 10^3 | <10^3 | Yes |  | Increase | Decrease | No |
|  |  |  | Cytomegalovirus | <10^3 | Not detected | No |  | Not detected | 10^3 | No |  | - | - | - |
|  |  |  | Pneumocystis jirovecii | Not detected | <10^3 | No |  | Not detected | Not detected | Yes |  | - | - | - |
|  |  |  | Human alphaherpesvirus 1 | Not detected | <10^3 | No |  | ≥10^6 | ≥10^6 | Yes |  | - | - | - |
|  |  |  | Klebsiella pneumoniae | Not detected | Not detected | Yes |  | 10^4 | 10^3 | Yes |  | - | - | - |
|  |  |  | Escherichia coli | Not detected | Not detected | Yes |  | 10^3 | Not detected | No |  | - | - | - |
|  |  |  | Elizabethkingia meningoseptica | Not detected | Not detected | Yes |  | <10^3 | 10^4 | Yes |  | - | - | - |
| P3 | 73 | Male | Influenza virus | ≥10^6 | ≥10^6 | Yes |  | 10^4 | 10^5 | Yes |  | Decrease | Decrease | Yes |
|  |  |  | Acinetobacter baumannii | ≥10^6 | ≥10^6 | Yes |  | ≥10^6 | ≥10^6 | Yes |  | No change | No change | Yes |
|  |  |  | Stenotrophomonas maltophilia | ≥10^6 | <10^3 | Yes |  | ≥10^6 | ≥10^6 | Yes |  | No change | Increase | No |
|  |  |  | Staphylococcus aureus | 10^4 | Not detected | No |  | 10^5 | <10^3 | Yes |  | - | - | - |
|  |  |  | Escherichia coli | <10^3 | <10^3 | Yes |  | ≥10^6 | <10^3 | Yes |  | Increase | No change | No |
|  |  |  | Klebsiella pneumoniae | <10^3 | <10^3 | Yes |  | 10^5 | Not detected | No |  | Increase | Not detected | No |
|  |  |  | Ureaplasma urealyticum | Not detected | <10^3 | No |  | Not detected | Not detected | Yes |  | - | - | - |
|  |  |  | Enterococcus faecalis | Not detected | Not detected | Yes |  | 10^5 | <10^3 | Yes |  | - | - | - |
|  |  |  | Enterococcus faecium | Not detected | Not detected | Yes |  | 10^3 | Not detected | No |  | - | - | - |
|  |  |  | Candida tropicalis | Not detected | Not detected | Yes |  | <10^3 | <10^3 | Yes |  | - | - | - |
|  |  |  | Candida albicans | Not detected | Not detected | Yes |  | <10^3 | <10^3 | Yes |  | - | - | - |
| P4 | 91 | Male | Haemophilus influenzae | 10^5 | 10^5 | Yes |  | - | - | - |  | - | - | - |
|  |  |  | Staphylococcus aureus | 10^5 | 10^3 | Yes |  | - | - | - |  | - | - | - |
|  |  |  | Human alphaherpesvirus 1 | <10^3 | 10^3 | Yes |  | - | - | - |  | - | - | - |
|  |  |  | Candida albicans | <10^3 | Not detected | No |  | - | - | - |  | - | - | - |
|  |  |  | Epstein-Barr virus | <10^3 | <10^3 | Yes |  | - | - | - |  | - | - | - |
| P5 | 78 | Male | Influenza virus | <10^3 | 10^5 | Yes |  | 10^4 | 10^3 | Yes |  | Increase | Decrease | No |
|  |  |  | Epstein-Barr virus | 10^3 | 10^5 | Yes |  | <10^3 | 10^5 | Yes |  | Decrease | No change | No |
|  |  |  | Enterococcus faecium | 10^3 | 10^3 | Yes |  | Not detected | Not detected | Yes |  | Not detected | Not detected | Yes |
|  |  |  | Aspergillus flavus | Not detected | <10^3 | No |  | Not detected | Not detected | Yes |  | - | - | - |
|  |  |  | Candida albicans | Not detected | 10^3 | No |  | Not detected | Not detected | Yes |  | - | - | - |
|  |  |  | Cytomegalovirus | Not detected | Not detected | Yes |  | Not detected | <10^3 | No |  | - | - | - |
| P6 | 87 | Female | Human alphaherpesvirus 1 | ≥10^6 | <10^3 | Yes |  | ≥10^6 | Not detected | No |  | No change | Not detected | No |
|  |  |  | Klebsiella pneumoniae | ≥10^6 | ≥10^6 | Yes |  | ≥10^6 | ≥10^6 | Yes |  | No change | No change | Yes |
|  |  |  | Enterococcus faecium | 10^3 | <10^3 | Yes |  | 10^3 | <10^3 | Yes |  | No change | No change | Yes |
|  |  |  | Candida albicans | <10^3 | 10^3 | Yes |  | <10^3 | <10^3 | Yes |  | No change | Decrease | No |
|  |  |  | Cytomegalovirus | <10^3 | <10^3 | Yes |  | Not detected | Not detected | Yes |  | Not detected | Not detected | Yes |
|  |  |  | Human parvovirus B19 | <10^3 | <10^3 | Yes |  | Not detected | Not detected | Yes |  | Not detected | Not detected | Yes |
|  |  |  | Aspergillus fumigatus | Not detected | <10^3 | No |  | Not detected | Not detected | Yes |  | - | - | - |
|  |  |  | Staphylococcus aureus | Not detected | Not detected | Yes |  | 10^3 | 10^4 | Yes |  | - | - | - |
| P7 | 85 | Male | Staphylococcus aureus | ≥10^6 | Not detected | No |  | - | - | - |  | - | - | - |
|  |  |  | Acinetobacter baumannii | ≥10^6 | 10^5 | Yes |  | - | - | - |  | - | - | - |
|  |  |  | Pseudomonas aeruginosa | ≥10^6 | <10^3 | Yes |  | - | - | - |  | - | - | - |
|  |  |  | Stenotrophomonas maltophilia | 10^5 | Not detected | No |  | - | - | - |  | - | - | - |
|  |  |  | Achromobacter xylosoxidans | 10^4 | Not detected | No |  | - | - | - |  | - | - | - |
|  |  |  | Enterococcus faecium | 10^4 | Not detected | No |  | - | - | - |  | - | - | - |
|  |  |  | Candida glabrata | 10^3 | Not detected | No |  | - | - | - |  | - | - | - |
|  |  |  | Epstein-Barr virus | <10^3 | Not detected | No |  | - | - | - |  | - | - | - |
| P8 | 79 | Female | Pseudomonas aeruginosa | ≥10^6 | ≥10^6 | Yes |  | ≥10^6 | ≥10^6 | Yes |  | No change | No change | Yes |
|  |  |  | Stenotrophomonas maltophilia | ≥10^6 | ≥10^6 | Yes |  | ≥10^6 | 10^5 | Yes |  | No change | Decrease | No |
|  |  |  | Achromobacter xylosoxidans | 10^4 | ≥10^6 | Yes |  | 10^5 | <10^3 | Yes |  | Increase | Decrease | No |
|  |  |  | Staphylococcus aureus | <10^3 | Not detected | No |  | 10^5 | Not detected | No |  | - | - | - |
|  |  |  | Acinetobacter baumannii | <10^3 | 10^3 | Yes |  | 10^3 | 10^3 | Yes |  | Increase | No change | No |
|  |  |  | Epstein-Barr virus | <10^3 | <10^3 | Yes |  | <10^3 | 10^3 | Yes |  | No change | Increase | No |
|  |  |  | Elizabethkingia anophelis | Not detected | <10^3 | No |  | <10^3 | 10^3 | Yes |  | - | - | - |
|  |  |  | Cytomegalovirus | Not detected | <10^3 | No |  | Not detected | 10^3 | No |  | - | - | - |
| P9 | 68 | Male | Pseudomonas aeruginosa | ≥10^6 | ≥10^6 | Yes |  | ≥10^6 | ≥10^6 | Yes |  | No change | No change | Yes |
|  |  |  | Streptococcus pneumoniae | 10^5 | <10^3 | Yes |  | <10^3 | 10^3 | Yes |  | Decrease | Increase | No |
|  |  |  | Candida auris | 10^5 | Not detected | No |  | 10^3 | <10^3 | Yes |  | - | - | - |
|  |  |  | Stenotrophomonas maltophilia | 10^5 | <10^3 | Yes |  | ≥10^6 | 10^3 | Yes |  | Increase | Increase | Yes |
|  |  |  | Achromobacter xylosoxidans | ≥10^6 | <10^3 | Yes |  | ≥10^6 | 10^3 | Yes |  | No change | Increase | No |
|  |  |  | Klebsiella pneumoniae | 10^3 | <10^3 | Yes |  | 10^3 | ≥10^6 | Yes |  | No change | Increase | No |
|  |  |  | Candida tropicalis | <10^3 | Not detected | No |  | <10^3 | <10^3 | Yes |  | - | - | - |
|  |  |  | Elizabethkingia anophelis | <10^3 | <10^3 | Yes |  | <10^3 | 10^3 | Yes |  | No change | Increase | No |
|  |  |  | Candida albicans | Not detected | <10^3 | No |  | <10^3 | 10^3 | Yes |  | - | - | - |
|  |  |  | Candida glabrata | Not detected | Not detected | Yes |  | <10^3 | 10^3 | Yes |  | - | - | - |
|  |  |  | Escherichia coli | Not detected | Not detected | Yes |  | <10^3 | ≥10^6 | Yes |  | - | - | - |
|  |  |  | Enterococcus faecium | Not detected | Not detected | Yes |  | Not detected | 10^3 | No |  | - | - | - |
|  |  |  | Epstein-Barr virus | Not detected | Not detected | Yes |  | Not detected | <10^3 | No |  | - | - | - |
| P10 | 69 | Female | Enterococcus faecium | <10^3 | ≥10^6 | Yes |  | 10^3 | 10^3 | Yes |  | Increase | Decrease | No |
|  |  |  | Epstein-Barr virus | <10^3 | 10^3 | Yes |  | 10^4 | 10^4 | Yes |  | Increase | Increase | Yes |
|  |  |  | Candida tropicalis | Not detected | ≥10^6 | No |  | 10^3 | 10^5 | Yes |  | - | - | - |
|  |  |  | Candida glabrata | Not detected | <10^3 | No |  | Not detected | <10^3 | No |  | - | - | - |
| P11 | 89 | Male | Staphylococcus aureus | ≥10^6 | ≥10^6 | Yes |  | ≥10^6 | 10^5 | Yes |  | No change | Decrease | No |
|  |  |  | Epstein-Barr virus | ≥10^6 | 10^3 | Yes |  | 10^3 | Not detected | No |  | Decrease | Not detected | Yes |
|  |  |  | Candida albicans | <10^3 | 10^3 | Yes |  | <10^3 | Not detected | No |  | No change | Not detected | No |
|  |  |  | Enterococcus faecium | <10^3 | 10^5 | Yes |  | <10^3 | Not detected | No |  | No change | Not detected | No |
|  |  |  | Klebsiella pneumoniae | Not detected | Not detected | Yes |  | ≥10^6 | ≥10^6 | Yes |  | - | - | - |
|  |  |  | Acinetobacter baumannii | Not detected | Not detected | Yes |  | ≥10^6 | 10^5 | Yes |  | - | - | - |
|  |  |  | Stenotrophomonas maltophilia | Not detected | Not detected | Yes |  | <10^3 | ≥10^6 | Yes |  | - | - | - |
|  |  |  | Cytomegalovirus | Not detected | Not detected | Yes |  | <10^3 | <10^3 | Yes |  | - | - | - |
| P12 | 60 | Male | Candida albicans | Not detected | 10^3 | No |  | <10^3 | Not detected | No |  | - | - | - |
|  |  |  | Staphylococcus aureus | Not detected | Not detected | Yes |  | ≥10^6 | 10^3 | Yes |  | - | - | - |
|  |  |  | Acinetobacter baumannii | Not detected | Not detected | Yes |  | ≥10^6 | ≥10^6 | Yes |  | - | - | - |
|  |  |  | Achromobacter xylosoxidans | Not detected | Not detected | Yes |  | ≥10^6 | 10^3 | Yes |  | - | - | - |
|  |  |  | Serratia marcescens | <10^3 | Not detected | No |  | Not detected | 10^3 | No |  | - | - | - |
|  |  |  | Stenotrophomonas maltophilia | <10^3 | Not detected | No |  | Not detected | ≥10^6 | No |  | - | - | - |
|  |  |  | Pseudomonas aeruginosa | Not detected | Not detected | Yes |  | Not detected | <10^3 | No |  | - | - | - |
| P13 | 59 | Male | Enterococcus faecium | ≥10^6 | 10^3 | Yes |  | - | - | - |  | - | - | - |
|  |  |  | Klebsiella pneumoniae | ≥10^6 | ≥10^6 | Yes |  | - | - | - |  | - | - | - |
|  |  |  | Stenotrophomonas maltophilia | ≥10^6 | ≥10^6 | Yes |  | - | - | - |  | - | - | - |
|  |  |  | Acinetobacter baumannii | ≥10^6 | ≥10^6 | Yes |  | - | - | - |  | - | - | - |
|  |  |  | Achromobacter xylosoxidans | ≥10^6 | 10^4 | Yes |  | - | - | - |  | - | - | - |
|  |  |  | Candida glabrata | <10^3 | Not detected | No |  | - | - | - |  | - | - | - |
|  |  |  | Cytomegalovirus | <10^3 | <10^3 | Yes |  | - | - | - |  | - | - | - |
|  |  |  | Epstein-Barr virus | <10^3 | <10^3 | Yes |  | - | - | - |  | - | - | - |
| P14 | 86 | Male | Enterococcus faecium | 10^5 | 10^3 | Yes |  | - | - | - |  | - | - | - |
|  |  |  | Escherichia coli | 10^5 | ≥10^6 | Yes |  | - | - | - |  | - | - | - |
|  |  |  | Rhinovirus | <10^3 | 10^3 | Yes |  | - | - | - |  | - | - | - |
|  |  |  | Pseudomonas aeruginosa | <10^3 | <10^3 | Yes |  | - | - | - |  | - | - | - |
|  |  |  | Candida parapsilosis | <10^3 | 10^3 | Yes |  | - | - | - |  | - | - | - |
|  |  |  | Klebsiella pneumoniae | <10^3 | <10^3 | Yes |  | - | - | - |  | - | - | - |
|  |  |  | Epstein-Barr virus | <10^3 | <10^3 | Yes |  | - | - | - |  | - | - | - |
| P15 | 41 | Female | Candida albicans | <10^3 | 10^3 | Yes |  | - | - | - |  | - | - | - |
|  |  |  | Epstein-Barr virus | <10^3 | <10^3 | Yes |  | - | - | - |  | - | - | - |
|  |  |  | Klebsiella pneumoniae | Not detected | 10^4 | No |  | - | - | - |  | - | - | - |
|  |  |  | Proteus mirabilis | Not detected | 10^3 | No |  | - | - | - |  | - | - | - |
|  |  |  | Acinetobacter baumannii | Not detected | <10^3 | No |  | - | - | - |  | - | - | - |
|  |  |  | Candida glabrata | Not detected | <10^3 | No |  | - | - | - |  | - | - | - |
|  |  |  | Staphylococcus aureus | Not detected | <10^3 | No |  | - | - | - |  | - | - | - |
|  |  |  | Enterococcus faecium | Not detected | <10^3 | No |  | - | - | - |  | - | - | - |
|  |  |  | Escherichia coli | Not detected | <10^3 | No |  | - | - | - |  | - | - | - |
|  |  |  | Cytomegalovirus | Not detected | <10^3 | No |  | - | - | - |  | - | - | - |
| P16 | 93 | Female | Pseudomonas aeruginosa | ≥10^6 | ≥10^6 | Yes |  | ≥10^6 | 10^5 | Yes |  | No change | Decrease | No |
|  |  |  | Proteus mirabilis | 10^5 | Not detected | No |  | ≥10^6 | Not detected | No |  | - | - | - |
|  |  |  | Candida auris | 10^4 | Not detected | No |  | Not detected | Not detected | Yes |  | - | - | - |
|  |  |  | Stenotrophomonas maltophilia | 10^3 | ≥10^6 | Yes |  | 10^3 | 10^3 | Yes |  | No change | Decrease | No |
|  |  |  | Acinetobacter baumannii | <10^3 | 10^3 | Yes |  | ≥10^6 | <10^3 | Yes |  | Increase | Decrease | No |
|  |  |  | Klebsiella pneumoniae | <10^3 | Not detected | No |  | ≥10^6 | ≥10^6 | Yes |  | - | - | - |
|  |  |  | Epstein-Barr virus | <10^3 | <10^3 | Yes |  | <10^3 | Not detected | No |  | No change | Not detected | No |
|  |  |  | Candida auris | Not detected | Not detected | Yes |  | <10^3 | Not detected | No |  | - | - | - |
| P17 | 65 | Male | Human respiratory syncytial virus | 10^5 | ≥10^6 | Yes |  | - | - | - |  | - | - | - |
|  |  |  | Epstein-Barr virus | 10^5 | <10^3 | Yes |  | - | - | - |  | - | - | - |
| P18 | 70 | Male | Haemophilus influenzae | 10^3 | 10^4 | Yes |  | - | - | - |  | - | - | - |
|  |  |  | Acinetobacter baumannii | 10^3 | Not detected | No |  | - | - | - |  | - | - | - |
|  |  |  | Candida tropicalis | 10^3 | Not detected | No |  | - | - | - |  | - | - | - |
|  |  |  | Streptococcus pneumoniae | 10^3 | 10^5 | Yes |  | - | - | - |  | - | - | - |
|  |  |  | Epstein-Barr virus | <10^3 | <10^3 | Yes |  | - | - | - |  | - | - | - |
| P19 | 77 | Male | Staphylococcus aureus | ≥10^6 | 10^4 | Yes |  | Not detected | 10^3 | No |  | Not detected | Decrease | Yes |
|  |  |  | Pseudomonas aeruginosa | ≥10^6 | 10^4 | Yes |  | <10^3 | 10^4 | Yes |  | Decrease | No change | No |
|  |  |  | Candida albicans | ≥10^6 | Not detected | No |  | Not detected | Not detected | Yes |  | - | - | - |
|  |  |  | Epstein-Barr virus | 10^3 | <10^3 | Yes |  | Not detected | <10^3 | No |  | Not detected | No change | No |
|  |  |  | Enterococcus faecium | 10^3 | <10^3 | Yes |  | Not detected | Not detected | Yes |  | Not detected | Not detected | Yes |
|  |  |  | Human alphaherpesvirus 1 | Not detected | 10^3 | No |  | Not detected | Not detected | Yes |  | - | - | - |
|  |  |  | Achromobacter xylosoxidans | Not detected | <10^3 | No |  | Not detected | <10^3 | No |  | - | - | - |
|  |  |  | Stenotrophomonas maltophilia | Not detected | <10^3 | No |  | Not detected | Not detected | Yes |  | - | - | - |
|  |  |  | Severe acute respiratory syndrome coronavirus 2 | Not detected | Not detected | Yes |  | ≥10^6 | 10^5 | Yes |  | - | - | - |
| P20 | 44 | Male | Enterococcus faecium | ≥10^6 | 10^5 | Yes |  | ≥10^6 | Not detected | No |  | No change | Not detected | No |
|  |  |  | Influenza virus | ≥10^6 | ≥10^6 | Yes |  | <10^3 | ≥10^6 | Yes |  | Decrease | No change | No |
|  |  |  | Severe acute respiratory syndrome coronavirus 2 | 10^4 | 10^4 | Yes |  | 10^4 | <10^3 | Yes |  | No change | Decrease | No |
|  |  |  | Stenotrophomonas maltophilia | Not detected | Not detected | Yes |  | ≥10^6 | ≥10^6 | Yes |  | - | - | - |
|  |  |  | Candida parapsilosis | Not detected | Not detected | Yes |  | <10^3 | 10^3 | Yes |  | - | - | - |
| P21 | 92 | Male | Pseudomonas aeruginosa | ≥10^6 | ≥10^6 | Yes |  | ≥10^6 | 10^5 | Yes |  | No change | Decrease | No |
|  |  |  | Stenotrophomonas maltophilia | 10^5 | <10^3 | Yes |  | 10^3 | 10^4 | Yes |  | Decrease | Increase | No |
|  |  |  | Staphylococcus aureus | 10^3 | 10^4 | Yes |  | ≥10^6 | ≥10^6 | Yes |  | Increase | Increase | Yes |
|  |  |  | Epstein-Barr virus | <10^3 | Not detected | No |  | <10^3 | Not detected | No |  | - | - | - |
|  |  |  | Enterococcus faecium | Not detected | 10^3 | No |  | 10^3 | <10^3 | Yes |  | - | - | - |
|  |  |  | Cytomegalovirus | Not detected | <10^3 | No |  | Not detected | Not detected | Yes |  | - | - | - |
| P22 | 85 | Male | Staphylococcus aureus | ≥10^6 | 10^5 | Yes |  | - | - | - |  | - | - | - |
|  |  |  | Human metapneumovirus | 10^5 | ≥10^6 | Yes |  | - | - | - |  | - | - | - |
|  |  |  | Proteus mirabilis | 10^5 | 10^5 | Yes |  | - | - | - |  | - | - | - |
|  |  |  | Haemophilus influenzae | 10^5 | ≥10^6 | Yes |  | - | - | - |  | - | - | - |
|  |  |  | Mycobacterium tuberculosis complex | <10^3 | <10^3 | Yes |  | - | - | - |  | - | - | - |
|  |  |  | Epstein-Barr virus | <10^3 | <10^3 | Yes |  | - | - | - |  | - | - | - |
| P23 | 64 | Male | Pseudomonas aeruginosa | 10^5 | ≥10^6 | Yes |  | ≥10^6 | ≥10^6 | Yes |  | Increase | No change | No |
|  |  |  | Klebsiella pneumoniae | 10^4 | ≥10^6 | Yes |  | 10^4 | ≥10^6 | Yes |  | No change | No change | Yes |
|  |  |  | Aspergillus fumigatus | <10^3 | <10^3 | Yes |  | 10^3 | <10^3 | Yes |  | Increase | No change | No |
|  |  |  | Streptococcus agalactiae | 10^4 | 10^3 | Yes |  | <10^3 | 10^4 | Yes |  | Decrease | Increase | No |
|  |  |  | Influenza virus | Not detected | <10^3 | No |  | Not detected | 10^5 | No |  | - | - | - |
|  |  |  | Acinetobacter baumannii | Not detected | Not detected | Yes |  | 10^5 | ≥10^6 | Yes |  | - | - | - |
|  |  |  | Enterococcus faecium | Not detected | Not detected | Yes |  | <10^3 | Not detected | No |  | - | - | - |
|  |  |  | Stenotrophomonas maltophilia | Not detected | Not detected | Yes |  | <10^3 | 10^5 | Yes |  | - | - | - |
|  |  |  | Enterobacter cloacae | Not detected | Not detected | Yes |  | Not detected | ≥10^6 | No |  | - | - | - |
| P24 | 88 | Male | Escherichia coli | ≥10^6 | ≥10^6 | Yes |  | 10^3 | Not detected | No |  | Decrease | Not detected | Yes |
|  |  |  | Staphylococcus aureus | ≥10^6 | Not detected | No |  | 10^3 | Not detected | No |  | - | - | - |
|  |  |  | Acinetobacter baumannii | ≥10^6 | 10^4 | Yes |  | ≥10^6 | 10^4 | Yes |  | No change | No change | Yes |
|  |  |  | Klebsiella pneumoniae | 10^5 | <10^3 | Yes |  | ≥10^6 | <10^3 | Yes |  | Increase | No change | No |
|  |  |  | Candida glabrata | Not detected | Not detected | Yes |  | <10^3 | Not detected | No |  | - | - | - |
| P25 | 84 | Male | Klebsiella pneumoniae | ≥10^6 | ≥10^6 | Yes |  | ≥10^6 | ≥10^6 | Yes |  | No change | No change | Yes |
|  |  |  | Stenotrophomonas maltophilia | ≥10^6 | 10^5 | Yes |  | ≥10^6 | 10^4 | Yes |  | No change | Decrease | No |
|  |  |  | Epstein-Barr virus | 10^5 | 10^4 | Yes |  | 10^3 | Not detected | No |  | Decrease | Not detected | Yes |
|  |  |  | Enterobacter cloacae | 10^5 | 10^3 | Yes |  | Not detected | Not detected | Yes |  | Not detected | Not detected | Yes |
|  |  |  | Acinetobacter baumannii | 10^4 | 10^3 | Yes |  | ≥10^6 | 10^5 | Yes |  | Increase | Increase | Yes |
|  |  |  | Candida auris | Not detected | Not detected | Yes |  | 10^5 | Not detected | No |  | - | - | - |
| P26 | 89 | Male | Pseudomonas aeruginosa | ≥10^6 | ≥10^6 | Yes |  | ≥10^6 | 10^4 | Yes |  | No change | Decrease | No |
|  |  |  | Acinetobacter baumannii | 10^5 | 10^5 | Yes |  | ≥10^6 | 10^5 | Yes |  | Increase | No change | No |
|  |  |  | Klebsiella pneumoniae | 10^5 | 10^3 | Yes |  | 10^5 | Not detected | No |  | No change | Not detected | No |
|  |  |  | Stenotrophomonas maltophilia | 10^4 | 10^5 | Yes |  | 10^5 | 10^5 | Yes |  | Increase | No change | No |
|  |  |  | Staphylococcus aureus | 10^4 | 10^4 | Yes |  | Not detected | Not detected | Yes |  | Not detected | Not detected | Yes |
|  |  |  | Achromobacter xylosoxidans | 10^5 | 10^4 | Yes |  | 10^5 | 10^3 | Yes |  | No change | Decrease | No |
|  |  |  | Candida albicans | 10^5 | 10^3 | Yes |  | <10^3 | <10^3 | Yes |  | Decrease | Decrease | Yes |
|  |  |  | Cytomegalovirus | 10^3 | <10^3 | Yes |  | <10^3 | Not detected | No |  | Decrease | Not detected | Yes |
|  |  |  | Escherichia coli | Not detected | 10^5 | No |  | Not detected | Not detected | Yes |  | - | - | - |
|  |  |  | Candida tropicalis | Not detected | Not detected | Yes |  | <10^3 | <10^3 | Yes |  | - | - | - |
|  |  |  | Elizabethkingia meningoseptica | Not detected | Not detected | Yes |  | Not detected | 10^5 | No |  | - | - | - |
| P27 | 61 | Male | Pseudomonas aeruginosa | ≥10^6 | 10^5 | Yes |  | 10^5 | 10^3 | Yes |  | Decrease | Decrease | Yes |
|  |  |  | Klebsiella pneumoniae | ≥10^6 | ≥10^6 | Yes |  | 10^5 | 10^5 | Yes |  | Decrease | Decrease | Yes |
|  |  |  | Stenotrophomonas maltophilia | ≥10^6 | 10^4 | Yes |  | ≥10^6 | 10^5 | Yes |  | No change | Increase | No |
|  |  |  | Acinetobacter baumannii | 10^3 | Not detected | No |  | Not detected | Not detected | Yes |  | - | - | - |
|  |  |  | Escherichia coli | <10^3 | Not detected | No |  | Not detected | Not detected | Yes |  | - | - | - |
|  |  |  | Cytomegalovirus | <10^3 | 10^3 | Yes |  | 10^4 | 10^3 | Yes |  | Increase | No change | No |
| P28 | 89 | Male | Influenza virus | ≥10^6 | 10^3 | Yes |  | <10^3 | 10^5 | Yes |  | Decrease | Increase | No |
|  |  |  | Acinetobacter baumannii | ≥10^6 | 10^5 | Yes |  | 10^5 | 10^4 | Yes |  | Decrease | Decrease | Yes |
|  |  |  | Staphylococcus aureus | ≥10^6 | <10^3 | Yes |  | 10^5 | 10^3 | Yes |  | Decrease | Increase | No |
|  |  |  | Human alphaherpesvirus 1 | Not detected | Not detected | Yes |  | ≥10^6 | <10^3 | Yes |  | - | - | - |
|  |  |  | Candida glabrata | Not detected | Not detected | Yes |  | 10^3 | <10^3 | Yes |  | - | - | - |
|  |  |  | Candida albicans | Not detected | Not detected | Yes |  | 10^3 | Not detected | No |  | - | - | - |
| P29 | 92 | Male | Mycoplasmoides pneumoniae | ≥10^6 | ≥10^6 | Yes |  | 10^3 | 10^3 | Yes |  | Decrease | Decrease | Yes |
|  |  |  | Staphylococcus aureus | 10^4 | <10^3 | Yes |  | 10^5 | 10^4 | Yes |  | Increase | Increase | Yes |
|  |  |  | Candida tropicalis | 10^3 | <10^3 | Yes |  | 10^4 | Not detected | No |  | Increase | Not detected | No |
|  |  |  | Acinetobacter baumannii | 10^3 | Not detected | No |  | 10^4 | 10^4 | Yes |  | - | - | - |
|  |  |  | Proteus mirabilis | Not detected | Not detected | Yes |  | ≥10^6 | Not detected | No |  | - | - | - |
|  |  |  | Human alphaherpesvirus 1 | Not detected | Not detected | Yes |  | 10^5 | Not detected | No |  | - | - | - |
|  |  |  | Pseudomonas aeruginosa | Not detected | Not detected | Yes |  | 10^4 | Not detected | No |  | - | - | - |
|  |  |  | Enterococcus faecium | Not detected | Not detected | Yes |  | 10^4 | Not detected | No |  | - | - | - |
|  |  |  | Stenotrophomonas maltophilia | Not detected | Not detected | Yes |  | 10^3 | 10^3 | Yes |  | - | - | - |
| P30 | 51 | Female | Pseudomonas aeruginosa | ≥10^6 | 10^5 | Yes |  | 10^5 | <10^3 | Yes |  | Decrease | Decrease | Yes |
|  |  |  | Stenotrophomonas maltophilia | ≥10^6 | 10^4 | Yes |  | 10^5 | 10^3 | Yes |  | Decrease | Decrease | Yes |
|  |  |  | Candida tropicalis | 10^4 | Not detected | No |  | 10^4 | Not detected | No |  | - | - | - |
|  |  |  | Candida albicans | 10^3 | 10^3 | Yes |  | 10^3 | <10^3 | Yes |  | No change | Decrease | No |
|  |  |  | Elizabethkingia anophelis | <10^3 | 10^3 | Yes |  | Not detected | <10^3 | No |  | Not detected | Decrease | Yes |
|  |  |  | Enterococcus faecalis | Not detected | Not detected | Yes |  | 10^3 | Not detected | No |  | - | - | - |
| P31 | 55 | Male | Pseudomonas aeruginosa | <10^3 | <10^3 | Yes |  | 10^3 | Not detected | No |  | Increase | Not detected | No |
|  |  |  | Acinetobacter baumannii | Not detected | Not detected | Yes |  | 10^5 | <10^3 | Yes |  | - | - | - |
| P32 | 47 | Male | Pseudomonas aeruginosa | 10^5 | 10^5 | Yes |  | 10^5 | 10^4 | Yes |  | No change | Decrease | No |
|  |  |  | Stenotrophomonas maltophilia | 10^5 | 10^4 | Yes |  | 10^4 | <10^3 | Yes |  | Decrease | Decrease | Yes |
|  |  |  | Mycobacteroides abscessus | 10^3 | <10^3 | Yes |  | 10^4 | <10^3 | Yes |  | Increase | No change | No |
|  |  |  | Achromobacter xylosoxidans | 10^4 | 10^4 | Yes |  | 10^5 | 10^3 | Yes |  | Increase | Decrease | No |
|  |  |  | Serratia marcescens | <10^3 | 10^4 | Yes |  | Not detected | 10^4 | No |  | Not detected | No change | No |
|  |  |  | Cytomegalovirus | Not detected | <10^3 | No |  | Not detected | Not detected | Yes |  | - | - | - |
|  |  |  | Klebsiella pneumoniae | Not detected | Not detected | Yes |  | 10^4 | <10^3 | Yes |  | - | - | - |
|  |  |  | Enterococcus faecium | Not detected | Not detected | Yes |  | 10^3 | Not detected | No |  | - | - | - |
|  |  |  | Candida albicans | Not detected | Not detected | Yes |  | <10^3 | Not detected | No |  | - | - | - |
| P33 | 75 | Female | Staphylococcus aureus | 10^4 | Not detected | No |  | - | - | - |  | - | - | - |
|  |  |  | Epstein-Barr virus | 10^3 | <10^3 | Yes |  | - | - | - |  | - | - | - |
|  |  |  | Cytomegalovirus | <10^3 | Not detected | No |  | - | - | - |  | - | - | - |
|  |  |  | Proteus mirabilis | Not detected | 10^4 | No |  | - | - | - |  | - | - | - |
| P34 | 96 | Male | Staphylococcus aureus | 10^5 | <10^3 | Yes |  | ≥10^6 | 10^3 | Yes |  | Increase | Increase | Yes |
|  |  |  | Epstein-Barr virus | 10^4 | Not detected | No |  | 10^3 | Not detected | No |  | - | - | - |
|  |  |  | Candida glabrata | <10^3 | Not detected | No |  | Not detected | Not detected | Yes |  | - | - | - |
|  |  |  | Stenotrophomonas maltophilia | Not detected | Not detected | Yes |  | ≥10^6 | <10^3 | Yes |  | - | - | - |
| P35 | 87 | Male | Human alphaherpesvirus 1 | ≥10^6 | 10^5 | Yes |  | 10^5 | 10^5 | Yes |  | Decrease | No change | No |
|  |  |  | Acinetobacter baumannii | 10^5 | 10^4 | Yes |  | 10^4 | 10^5 | Yes |  | Decrease | Increase | No |
|  |  |  | Epstein-Barr virus | 10^5 | Not detected | No |  | <10^3 | <10^3 | Yes |  | - | - | - |
|  |  |  | Human respiratory syncytial virus | 10^4 | ≥10^6 | Yes |  | Not detected | 10^4 | No |  | Not detected | Decrease | Yes |
|  |  |  | Enterococcus faecium | 10^5 | Not detected | No |  | <10^3 | <10^3 | Yes |  | - | - | - |
|  |  |  | Cytomegalovirus | 10^3 | <10^3 | Yes |  | 10^3 | <10^3 | Yes |  | No change | No change | Yes |
|  |  |  | Stenotrophomonas maltophilia | 10^3 | 10^5 | Yes |  | 10^3 | 10^5 | Yes |  | No change | No change | Yes |
|  |  |  | Candida tropicalis | Not detected | Not detected | Yes |  | Not detected | 10^3 | No |  | - | - | - |
| P36 | 80 | Male | Severe acute respiratory syndrome coronavirus 2 | ≥10^6 | 10^5 | Yes |  | - | - | - |  | - | - | - |
|  |  |  | Staphylococcus aureus | 10^5 | 10^3 | Yes |  | - | - | - |  | - | - | - |
|  |  |  | Klebsiella pneumoniae | 10^4 | 10^4 | Yes |  | - | - | - |  | - | - | - |
|  |  |  | Pneumocystis jirovecii | 10^3 | 10^3 | Yes |  | - | - | - |  | - | - | - |
|  |  |  | Candida glabrata | 10^3 | 10^4 | Yes |  | - | - | - |  | - | - | - |
| P37 | 89 | Female | Pseudomonas aeruginosa | 10^5 | 10^4 | Yes |  | 10^4 | 10^4 | Yes |  | Decrease | No change | No |
|  |  |  | Staphylococcus aureus | 10^5 | 10^4 | Yes |  | 10^5 | 10^4 | Yes |  | No change | No change | Yes |
|  |  |  | Stenotrophomonas maltophilia | 10^5 | <10^3 | Yes |  | 10^4 | <10^3 | Yes |  | Decrease | No change | No |
|  |  |  | Proteus mirabilis | 10^4 | <10^3 | Yes |  | Not detected | Not detected | Yes |  | Not detected | Not detected | Yes |
|  |  |  | Candida albicans | 10^4 | Not detected | No |  | 10^4 | 10^3 | Yes |  | - | - | - |
|  |  |  | Achromobacter xylosoxidans | 10^3 | <10^3 | Yes |  | Not detected | Not detected | Yes |  | Not detected | Not detected | Yes |
|  |  |  | Human alphaherpesvirus 1 | 10^3 | <10^3 | Yes |  | Not detected | Not detected | Yes |  | Not detected | Not detected | Yes |
|  |  |  | Epstein-Barr virus | 10^3 | 10^3 | Yes |  | 10^3 | 10^3 | Yes |  | No change | No change | Yes |
| P38 | 83 | Female | Pseudomonas aeruginosa | 10^5 | 10^5 | Yes |  | 10^5 | <10^3 | Yes |  | No change | Decrease | No |
|  |  |  | Enterobacter cloacae | 10^3 | Not detected | No |  | Not detected | Not detected | Yes |  | - | - | - |
|  |  |  | Enterococcus faecalis | Not detected | 10^3 | No |  | 10^3 | <10^3 | Yes |  | - | - | - |
|  |  |  | Candida albicans | Not detected | Not detected | Yes |  | 10^3 | Not detected | No |  | - | - | - |
| P39 | 64 | Male | Staphylococcus aureus | ≥10^6 | ≥10^6 | Yes |  | 10^4 | <10^3 | Yes |  | Decrease | Decrease | Yes |
|  |  |  | Pseudomonas aeruginosa | ≥10^6 | ≥10^6 | Yes |  | 10^4 | <10^3 | Yes |  | Decrease | Decrease | Yes |
|  |  |  | Streptococcus agalactiae | 10^5 | ≥10^6 | Yes |  | Not detected | <10^3 | No |  | Not detected | Decrease | Yes |
|  |  |  | Escherichia coli | 10^3 | ≥10^6 | Yes |  | 10^3 | <10^3 | Yes |  | No change | Decrease | No |
|  |  |  | Klebsiella pneumoniae | 10^3 | 10^5 | Yes |  | 10^4 | <10^3 | Yes |  | Increase | Decrease | No |
|  |  |  | Achromobacter xylosoxidans | 10^3 | 10^3 | Yes |  | 10^4 | Not detected | No |  | Increase | Not detected | No |
|  |  |  | Stenotrophomonas maltophilia | <10^3 | 10^3 | Yes |  | 10^5 | <10^3 | Yes |  | Increase | Decrease | No |
|  |  |  | Enterobacter cloacae | Not detected | 10^3 | No |  | Not detected | Not detected | Yes |  | - | - | - |
|  |  |  | Candida tropicalis | Not detected | <10^3 | No |  | Not detected | Not detected | Yes |  | - | - | - |
|  |  |  | Enterococcus faecium | Not detected | Not detected | Yes |  | 10^3 | Not detected | No |  | - | - | - |
|  |  |  | Human alphaherpesvirus 1 | Not detected | Not detected | Yes |  | 10^3 | 10^3 | Yes |  | - | - | - |
|  |  |  | Epstein-Barr virus | Not detected | Not detected | Yes |  | <10^3 | <10^3 | Yes |  | - | - | - |
| P40 | 85 | Male | Staphylococcus aureus | ≥10^6 | 10^3 | Yes |  | 10^5 | ≥10^6 | Yes |  | Decrease | Increase | No |
|  |  |  | Proteus mirabilis | 10^5 | ≥10^6 | Yes |  | ≥10^6 | <10^3 | Yes |  | Increase | Decrease | No |
|  |  |  | Escherichia coli | 10^4 | 10^4 | Yes |  | 10^5 | Not detected | No |  | Increase | Not detected | No |
|  |  |  | Candida parapsilosis | 10^3 | <10^3 | Yes |  | <10^3 | Not detected | No |  | Decrease | Not detected | Yes |
|  |  |  | Epstein-Barr virus | 10^3 | 10^3 | Yes |  | 10^3 | 10^3 | Yes |  | No change | No change | Yes |
|  |  |  | Ureaplasma urealyticum | <10^3 | 10^3 | Yes |  | Not detected | <10^3 | No |  | Not detected | Decrease | Yes |
|  |  |  | Pneumocystis jirovecii | <10^3 | <10^3 | Yes |  | Not detected | Not detected | Yes |  | Not detected | Not detected | Yes |
|  |  |  | Klebsiella pneumoniae | Not detected | Not detected | Yes |  | <10^3 | <10^3 | Yes |  | - | - | - |
| P41 | 84 | Male | Klebsiella pneumoniae | 10^5 | 10^5 | Yes |  | - | - | - |  | - | - | - |
|  |  |  | Acinetobacter baumannii | 10^5 | 10^5 | Yes |  | - | - | - |  | - | - | - |
|  |  |  | Human alphaherpesvirus 1 | 10^4 | Not detected | No |  | - | - | - |  | - | - | - |
|  |  |  | Influenza virus | 10^3 | 10^3 | Yes |  | - | - | - |  | - | - | - |
|  |  |  | Candida parapsilosis | 10^4 | Not detected | No |  | - | - | - |  | - | - | - |
| P42 | 89 | Female | Staphylococcus aureus | ≥10^6 | Not detected | No |  | ≥10^6 | <10^3 | Yes |  | - | - | - |
|  |  |  | Enterobacter cloacae | 10^3 | Not detected | No |  | ≥10^6 | Not detected | No |  | - | - | - |
|  |  |  | Epstein-Barr virus | <10^3 | Not detected | No |  | 10^5 | <10^3 | Yes |  | - | - | - |
|  |  |  | Escherichia coli | Not detected | Not detected | Yes |  | ≥10^6 | Not detected | No |  | - | - | - |
|  |  |  | Acinetobacter baumannii | Not detected | Not detected | Yes |  | 10^5 | 10^4 | Yes |  | - | - | - |
|  |  |  | Klebsiella pneumoniae | Not detected | Not detected | Yes |  | 10^5 | <10^3 | Yes |  | - | - | - |
|  |  |  | Pseudomonas aeruginosa | Not detected | Not detected | Yes |  | Not detected | <10^3 | No |  | - | - | - |
| P43 | 90 | Male | Klebsiella pneumoniae | 10^5 | 10^4 | Yes |  | <10^3 | 10^4 | Yes |  | Decrease | No change | No |
|  |  |  | Human respiratory syncytial virus | 10^3 | <10^3 | Yes |  | 10^4 | <10^3 | Yes |  | Increase | No change | No |
|  |  |  | Escherichia coli | 10^3 | Not detected | No |  | Not detected | Not detected | Yes |  | - | - | - |
|  |  |  | Epstein-Barr virus | 10^3 | <10^3 | Yes |  | 10^3 | 10^4 | Yes |  | No change | Increase | No |
|  |  |  | Enterococcus faecium | Not detected | Not detected | Yes |  | 10^4 | Not detected | No |  | - | - | - |
| P44 | 74 | Male | Staphylococcus aureus | 10^5 | ≥10^6 | Yes |  | ≥10^6 | 10^4 | Yes |  | Increase | Decrease | No |
|  |  |  | Influenza virus | <10^3 | 10^4 | Yes |  | <10^3 | 10^3 | Yes |  | No change | Decrease | No |
|  |  |  | Klebsiella pneumoniae | 10^3 | 10^5 | Yes |  | ≥10^6 | 10^4 | Yes |  | Increase | Decrease | No |
|  |  |  | Epstein-Barr virus | Not detected | Not detected | Yes |  | 10^4 | <10^3 | Yes |  | - | - | - |
| P45 | 73 | Male | Staphylococcus aureus | 10^5 | 10^5 | Yes |  | ≥10^6 | 10^5 | Yes |  | Increase | No change | No |
|  |  |  | Acinetobacter baumannii | 10^5 | 10^4 | Yes |  | 10^5 | 10^5 | Yes |  | No change | Increase | No |
|  |  |  | Stenotrophomonas maltophilia | 10^5 | ≥10^6 | Yes |  | 10^5 | 10^5 | Yes |  | No change | Decrease | No |
|  |  |  | Enterococcus faecium | 10^5 | 10^3 | Yes |  | 10^5 | ≥10^6 | Yes |  | No change | Increase | No |
|  |  |  | Klebsiella pneumoniae | 10^4 | 10^3 | Yes |  | 10^4 | 10^4 | Yes |  | No change | Increase | No |
|  |  |  | Severe acute respiratory syndrome coronavirus 2 | 10^3 | <10^3 | Yes |  | <10^3 | 10^3 | Yes |  | Decrease | Increase | No |
|  |  |  | Aspergillus fumigatus | <10^3 | Not detected | No |  | Not detected | <10^3 | No |  | - | - | - |
|  |  |  | Human alphaherpesvirus 1 | 10^3 | 10^5 | Yes |  | 10^4 | 10^4 | Yes |  | Increase | Decrease | No |
|  |  |  | Candida parapsilosis | Not detected | <10^3 | No |  | 10^3 | Not detected | No |  | - | - | - |
|  |  |  | Epstein-Barr virus | Not detected | Not detected | Yes |  | Not detected | 10^4 | No |  | - | - | - |
| P46 | 63 | Female | Acinetobacter baumannii | 10^5 | 10^4 | Yes |  | 10^5 | 10^4 | Yes |  | No change | No change | Yes |
|  |  |  | Staphylococcus aureus | 10^4 | Not detected | No |  | 10^5 | 10^5 | Yes |  | - | - | - |
|  |  |  | Escherichia coli | 10^4 | Not detected | No |  | ≥10^6 | 10^3 | Yes |  | - | - | - |
|  |  |  | Enterococcus faecalis | 10^3 | Not detected | No |  | 10^4 | Not detected | No |  | - | - | - |
|  |  |  | Epstein-Barr virus | 10^3 | <10^3 | Yes |  | Not detected | Not detected | Yes |  | Not detected | Not detected | Yes |
|  |  |  | Klebsiella pneumoniae | Not detected | <10^3 | No |  | Not detected | Not detected | Yes |  | - | - | - |
|  |  |  | Stenotrophomonas maltophilia | Not detected | Not detected | Yes |  | ≥10^6 | 10^4 | Yes |  | - | - | - |
|  |  |  | Candida albicans | Not detected | Not detected | Yes |  | 10^4 | Not detected | No |  | - | - | - |
|  |  |  | Human alphaherpesvirus 1 | Not detected | Not detected | Yes |  | 10^3 | 10^3 | Yes |  | - | - | - |
| P47 | 80 | Female | Klebsiella pneumoniae | 10^5 | 10^4 | Yes |  | <10^3 | 10^4 | Yes |  | Decrease | No change | No |
|  |  |  | Epstein-Barr virus | 10^5 | Not detected | No |  | <10^3 | 10^3 | Yes |  | - | - | - |
|  |  |  | Enterococcus faecalis | 10^4 | Not detected | No |  | Not detected | Not detected | Yes |  | - | - | - |
|  |  |  | Candida albicans | 10^3 | <10^3 | Yes |  | Not detected | Not detected | Yes |  | Not detected | Not detected | Yes |
|  |  |  | Human alphaherpesvirus 1 | 10^3 | 10^4 | Yes |  | 10^4 | 10^5 | Yes |  | Increase | Increase | Yes |
|  |  |  | Cytomegalovirus | Not detected | Not detected | Yes |  | 10^3 | 10^3 | Yes |  | - | - | - |
| P48 | 81 | Male | Pseudomonas aeruginosa | ≥10^6 | <10^3 | Yes |  | 10^3 | 10^3 | Yes |  | Decrease | Increase | No |
|  |  |  | Klebsiella pneumoniae | ≥10^6 | 10^5 | Yes |  | 10^4 | 10^4 | Yes |  | Decrease | Decrease | Yes |
|  |  |  | Achromobacter xylosoxidans | ≥10^6 | <10^3 | Yes |  | 10^4 | 10^4 | Yes |  | Decrease | Increase | No |
|  |  |  | Stenotrophomonas maltophilia | 10^5 | 10^4 | Yes |  | Not detected | 10^5 | No |  | Not detected | Increase | No |
| P49 | 79 | Female | Influenza virus | <10^3 | 10^5 | Yes |  | Not detected | 10^5 | No |  | Not detected | No change | No |
|  |  |  | Enterococcus faecium | 10^4 | <10^3 | Yes |  | ≥10^6 | Not detected | No |  | Increase | Not detected | No |
|  |  |  | Cytomegalovirus | 10^3 | <10^3 | Yes |  | Not detected | Not detected | Yes |  | Not detected | Not detected | Yes |
|  |  |  | Pneumocystis jirovecii | <10^3 | <10^3 | Yes |  | Not detected | Not detected | Yes |  | Not detected | Not detected | Yes |
|  |  |  | Stenotrophomonas maltophilia | Not detected | 10^5 | No |  | ≥10^6 | 10^5 | Yes |  | - | - | - |
|  |  |  | Pseudomonas aeruginosa | Not detected | 10^5 | No |  | Not detected | Not detected | Yes |  | - | - | - |
|  |  |  | Achromobacter xylosoxidans | Not detected | 10^5 | No |  | Not detected | <10^3 | No |  | - | - | - |
|  |  |  | Klebsiella pneumoniae | Not detected | 10^4 | No |  | 10^4 | Not detected | No |  | - | - | - |
|  |  |  | Human alphaherpesvirus 1 | Not detected | Not detected | Yes |  | 10^5 | Not detected | No |  | - | - | - |
|  |  |  | Epstein-Barr virus | Not detected | Not detected | Yes |  | 10^3 | <10^3 | Yes |  | - | - | - |
| P50 | 65 | Male | Enterococcus faecium | 10^5 | <10^3 | Yes |  | Not detected | Not detected | Yes |  | Not detected | Not detected | Yes |
|  |  |  | Acinetobacter baumannii | 10^4 | 10^3 | Yes |  | Not detected | Not detected | Yes |  | Not detected | Not detected | Yes |
|  |  |  | Stenotrophomonas maltophilia | 10^3 | <10^3 | Yes |  | 10^5 | 10^3 | Yes |  | Increase | Increase | Yes |
|  |  |  | Klebsiella pneumoniae | 10^3 | <10^3 | Yes |  | 10^4 | 10^3 | Yes |  | Increase | Increase | Yes |
|  |  |  | Candida glabrata | <10^3 | Not detected | No |  | Not detected | Not detected | Yes |  | - | - | - |
|  |  |  | Candida parapsilosis | <10^3 | Not detected | No |  | 10^3 | Not detected | No |  | - | - | - |
|  |  |  | Epstein-Barr virus | <10^3 | 10^3 | Yes |  | Not detected | Not detected | Yes |  | Not detected | Not detected | Yes |
|  |  |  | Human parvovirus B19 | <10^3 | <10^3 | Yes |  | Not detected | Not detected | Yes |  | Not detected | Not detected | Yes |
|  |  |  | Influenza virus | Not detected | 10^4 | No |  | <10^3 | 10^3 | Yes |  | - | - | - |
|  |  |  | Aspergillus fumigatus | Not detected | <10^3 | No |  | Not detected | <10^3 | No |  | - | - | - |
| P51 | 66 | Male | Streptococcus pneumoniae | <10^3 | <10^3 | Yes |  | Not detected | Not detected | Yes |  | Not detected | Not detected | Yes |
|  |  |  | Staphylococcus aureus | Not detected | <10^3 | No |  | Not detected | Not detected | Yes |  | - | - | - |
|  |  |  | Candida parapsilosis | Not detected | <10^3 | No |  | Not detected | <10^3 | No |  | - | - | - |
|  |  |  | Stenotrophomonas maltophilia | Not detected | Not detected | Yes |  | <10^3 | <10^3 | Yes |  | - | - | - |
| P52 | 93 | Female | Enterococcus faecium | 10^5 | <10^3 | Yes |  | 10^4 | Not detected | No |  | Decrease | Not detected | Yes |
|  |  |  | Staphylococcus aureus | 10^5 | <10^3 | Yes |  | 10^4 | Not detected | No |  | Decrease | Not detected | Yes |
|  |  |  | Epstein-Barr virus | 10^4 | 10^3 | Yes |  | 10^4 | 10^4 | Yes |  | No change | Increase | No |
|  |  |  | Influenza virus | 10^3 | 10^4 | Yes |  | Not detected | Not detected | Yes |  | Not detected | Not detected | Yes |
|  |  |  | Candida albicans | 10^5 | 10^4 | Yes |  | 10^3 | Not detected | No |  | Decrease | Not detected | Yes |
|  |  |  | Escherichia coli | 10^3 | 10^5 | Yes |  | Not detected | Not detected | Yes |  | Not detected | Not detected | Yes |
|  |  |  | Klebsiella pneumoniae | <10^3 | <10^3 | Yes |  | 10^5 | ≥10^6 | Yes |  | Increase | Increase | Yes |
|  |  |  | Stenotrophomonas maltophilia | Not detected | Not detected | Yes |  | 10^5 | 10^5 | Yes |  | - | - | - |
| P53 | 70 | Male | Enterococcus faecium | ≥10^6 | 10^3 | Yes |  | 10^4 | <10^3 | Yes |  | Decrease | Decrease | Yes |
|  |  |  | Human alphaherpesvirus 1 | 10^5 | <10^3 | Yes |  | 10^3 | Not detected | No |  | Decrease | Not detected | Yes |
|  |  |  | Pseudomonas aeruginosa | 10^5 | 10^3 | Yes |  | 10^5 | 10^4 | Yes |  | No change | Increase | No |
|  |  |  | Staphylococcus aureus | 10^4 | Not detected | No |  | Not detected | Not detected | Yes |  | - | - | - |
|  |  |  | Epstein-Barr virus | 10^4 | <10^3 | Yes |  | <10^3 | <10^3 | Yes |  | Decrease | No change | No |
|  |  |  | Candida albicans | 10^4 | Not detected | No |  | Not detected | Not detected | Yes |  | - | - | - |
|  |  |  | Acinetobacter baumannii | 10^3 | <10^3 | Yes |  | 10^5 | Not detected | No |  | Increase | Not detected | No |
|  |  |  | Candida parapsilosis | 10^3 | <10^3 | Yes |  | Not detected | Not detected | Yes |  | Not detected | Not detected | Yes |
|  |  |  | Cytomegalovirus | 10^3 | <10^3 | Yes |  | Not detected | 10^3 | No |  | Not detected | Increase | No |
| P54 | 85 | Male | Staphylococcus aureus | ≥10^6 | 10^5 | Yes |  | - | - | - |  | - | - | - |
|  |  |  | Severe acute respiratory syndrome coronavirus 2 | 10^3 | 10^5 | Yes |  | - | - | - |  | - | - | - |
|  |  |  | Candida albicans | 10^4 | 10^3 | Yes |  | - | - | - |  | - | - | - |
|  |  |  | Epstein-Barr virus | Not detected | 10^4 | No |  | - | - | - |  | - | - | - |
| P55 | 71 | Male | Stenotrophomonas maltophilia | ≥10^6 | 10^5 | Yes |  | - | - | - |  | - | - | - |
|  |  |  | Pseudomonas aeruginosa | ≥10^6 | 10^5 | Yes |  | - | - | - |  | - | - | - |
|  |  |  | Staphylococcus aureus | 10^5 | 10^4 | Yes |  | - | - | - |  | - | - | - |
|  |  |  | Klebsiella pneumoniae | 10^5 | ≥10^6 | Yes |  | - | - | - |  | - | - | - |
|  |  |  | Escherichia coli | 10^4 | 10^5 | Yes |  | - | - | - |  | - | - | - |
|  |  |  | Candida albicans | 10^4 | 10^3 | Yes |  | - | - | - |  | - | - | - |
|  |  |  | Achromobacter xylosoxidans | 10^4 | <10^3 | Yes |  | - | - | - |  | - | - | - |
|  |  |  | Epstein-Barr virus | 10^3 | Not detected | No |  | - | - | - |  | - | - | - |
|  |  |  | Cytomegalovirus | <10^3 | Not detected | No |  | - | - | - |  | - | - | - |
| P56 | 56 | Male | Pseudomonas aeruginosa | 10^5 | 10^5 | Yes |  | ≥10^6 | 10^4 | Yes |  | Increase | Decrease | No |
|  |  |  | Stenotrophomonas maltophilia | 10^5 | 10^5 | Yes |  | 10^5 | 10^4 | Yes |  | No change | Decrease | No |
|  |  |  | Enterobacter cloacae | 10^3 | Not detected | No |  | Not detected | Not detected | Yes |  | - | - | - |
|  |  |  | Candida tropicalis | 10^3 | 10^4 | Yes |  | 10^3 | <10^3 | Yes |  | No change | Decrease | No |
|  |  |  | Achromobacter xylosoxidans | Not detected | Not detected | Yes |  | 10^5 | Not detected | No |  | - | - | - |
|  |  |  | Enterococcus faecium | Not detected | Not detected | Yes |  | 10^4 | 10^4 | Yes |  | - | - | - |
|  |  |  | Klebsiella pneumoniae | Not detected | Not detected | Yes |  | Not detected | <10^3 | No |  | - | - | - |
| P57 | 94 | Male | Enterococcus faecium | ≥10^6 | <10^3 | Yes |  | Not detected | Not detected | Yes |  | Not detected | Not detected | Yes |
|  |  |  | Klebsiella pneumoniae | ≥10^6 | 10^4 | Yes |  | 10^5 | 10^5 | Yes |  | Decrease | Increase | No |
|  |  |  | Candida albicans | ≥10^6 | Not detected | No |  | 10^3 | 10^3 | Yes |  | - | - | - |
|  |  |  | Epstein-Barr virus | 10^5 | <10^3 | Yes |  | 10^4 | Not detected | No |  | Decrease | Not detected | Yes |
|  |  |  | Severe acute respiratory syndrome coronavirus 2 | 10^4 | 10^5 | Yes |  | <10^3 | 10^5 | Yes |  | Decrease | No change | No |
|  |  |  | Human alphaherpesvirus 1 | Not detected | Not detected | Yes |  | 10^5 | Not detected | No |  | - | - | - |
|  |  |  | Escherichia coli | Not detected | Not detected | Yes |  | 10^4 | Not detected | No |  | - | - | - |
|  |  |  | Candida auris | Not detected | Not detected | Yes |  | <10^3 | 10^4 | Yes |  | - | - | - |
|  |  |  | Pseudomonas aeruginosa | Not detected | Not detected | Yes |  | Not detected | 10^5 | No |  | - | - | - |
|  |  |  | Stenotrophomonas maltophilia | Not detected | Not detected | Yes |  | Not detected | 10^4 | No |  | - | - | - |
|  |  |  | Achromobacter xylosoxidans | Not detected | Not detected | Yes |  | Not detected | 10^3 | No |  | - | - | - |
| P58 | 84 | Female | Candida albicans | ≥10^6 | Not detected | No |  | - | - | - |  | - | - | - |
|  |  |  | Epstein-Barr virus | 10^5 | Not detected | No |  | - | - | - |  | - | - | - |
|  |  |  | Human alphaherpesvirus 1 | 10^5 | 10^4 | Yes |  | - | - | - |  | - | - | - |
|  |  |  | Enterococcus faecium | 10^4 | Not detected | No |  | - | - | - |  | - | - | - |
| P59 | 69 | Female | Escherichia coli | ≥10^6 | 10^5 | Yes |  | 10^4 | Not detected | No |  | Decrease | Not detected | Yes |
|  |  |  | Staphylococcus aureus | ≥10^6 | 10^3 | Yes |  | 10^5 | Not detected | No |  | Decrease | Not detected | Yes |
|  |  |  | Pseudomonas aeruginosa | ≥10^6 | 10^4 | Yes |  | ≥10^6 | ≥10^6 | Yes |  | No change | Increase | No |
|  |  |  | Klebsiella pneumoniae | ≥10^6 | 10^5 | Yes |  | 10^4 | <10^3 | Yes |  | Decrease | Decrease | Yes |
|  |  |  | Streptococcus agalactiae | <10^3 | 10^3 | Yes |  | Not detected | Not detected | Yes |  | Not detected | Not detected | Yes |
|  |  |  | Human alphaherpesvirus 1 | Not detected | Not detected | Yes |  | 10^5 | 10^4 | Yes |  | - | - | - |
|  |  |  | Epstein-Barr virus | Not detected | Not detected | Yes |  | 10^4 | 10^3 | Yes |  | - | - | - |
| P60 | 84 | Female | Stenotrophomonas maltophilia | 10^4 | 10^5 | Yes |  | <10^3 | 10^5 | Yes |  | Decrease | No change | No |
|  |  |  | Epstein-Barr virus | 10^3 | Not detected | No |  | 10^3 | Not detected | No |  | - | - | - |
|  |  |  | Cytomegalovirus | 10^3 | 10^3 | Yes |  | <10^3 | 10^4 | Yes |  | Decrease | Increase | No |
|  |  |  | Candida albicans | 10^3 | 10^5 | Yes |  | <10^3 | 10^3 | Yes |  | Decrease | Decrease | Yes |
|  |  |  | Acinetobacter baumannii | <10^3 | 10^4 | Yes |  | 10^3 | ≥10^6 | Yes |  | Increase | Increase | Yes |
|  |  |  | Pseudomonas aeruginosa | Not detected | 10^3 | No |  | Not detected | 10^4 | No |  | - | - | - |
|  |  |  | Corynebacterium striatum | Not detected | Not detected | Yes |  | 10^4 | ≥10^6 | Yes |  | - | - | - |
|  |  |  | Klebsiella pneumoniae | Not detected | Not detected | Yes |  | 10^4 | Not detected | No |  | - | - | - |
| P61 | 78 | Female | Stenotrophomonas maltophilia | 10^3 | 10^5 | Yes |  | Not detected | 10^5 | No |  | Not detected | No change | No |
|  |  |  | Achromobacter xylosoxidans | 10^3 | 10^4 | Yes |  | 10^3 | 10^5 | Yes |  | No change | Increase | No |
|  |  |  | Staphylococcus aureus | 10^3 | Not detected | No |  | Not detected | Not detected | Yes |  | - | - | - |
|  |  |  | Pseudomonas aeruginosa | <10^3 | 10^5 | Yes |  | 10^3 | ≥10^6 | Yes |  | Increase | Increase | Yes |
|  |  |  | Elizabethkingia meningoseptica | <10^3 | 10^3 | Yes |  | Not detected | 10^5 | No |  | Not detected | Increase | No |
|  |  |  | Klebsiella pneumoniae | Not detected | Not detected | Yes |  | 10^3 | ≥10^6 | Yes |  | - | - | - |
|  |  |  | Candida auris | Not detected | Not detected | Yes |  | 10^3 | 10^3 | Yes |  | - | - | - |
|  |  |  | Corynebacterium striatum | Not detected | Not detected | Yes |  | 10^3 | Not detected | No |  | - | - | - |
|  |  |  | Cytomegalovirus | Not detected | Not detected | Yes |  | <10^3 | 10^4 | Yes |  | - | - | - |
| P62 | 87 | Female | Proteus mirabilis | ≥10^6 | 10^3 | Yes |  | 10^4 | Not detected | No |  | Decrease | Not detected | Yes |
|  |  |  | Pseudomonas aeruginosa | 10^5 | 10^5 | Yes |  | ≥10^6 | ≥10^6 | Yes |  | Increase | Increase | Yes |
|  |  |  | Klebsiella pneumoniae | 10^5 | 10^4 | Yes |  | 10^4 | ≥10^6 | Yes |  | Decrease | Increase | No |
|  |  |  | Acinetobacter baumannii | 10^5 | 10^4 | Yes |  | 10^5 | ≥10^6 | Yes |  | No change | Increase | No |
|  |  |  | Cytomegalovirus | 10^4 | Not detected | No |  | <10^3 | 10^4 | Yes |  | - | - | - |
|  |  |  | Stenotrophomonas maltophilia | 10^4 | ≥10^6 | Yes |  | Not detected | Not detected | Yes |  | Not detected | Not detected | Yes |
|  |  |  | Elizabethkingia anophelis | <10^3 | 10^3 | Yes |  | 10^5 | 10^5 | Yes |  | Increase | Increase | Yes |
|  |  |  | Enterococcus faecium | Not detected | Not detected | Yes |  | <10^3 | Not detected | No |  | - | - | - |
|  |  |  | Human alphaherpesvirus 1 | Not detected | Not detected | Yes |  | Not detected | 10^4 | No |  | - | - | - |
| P63 | 80 | Female | Human alphaherpesvirus 1 | 10^4 | 10^3 | Yes |  | - | - | - |  | - | - | - |
|  |  |  | Klebsiella pneumoniae | 10^4 | <10^3 | Yes |  | - | - | - |  | - | - | - |
|  |  |  | Epstein-Barr virus | 10^4 | Not detected | No |  | - | - | - |  | - | - | - |
|  |  |  | Candida albicans | 10^5 | <10^3 | Yes |  | - | - | - |  | - | - | - |
|  |  |  | Enterococcus faecalis | 10^3 | Not detected | No |  | - | - | - |  | - | - | - |
|  |  |  | Acinetobacter baumannii | <10^3 | <10^3 | Yes |  | - | - | - |  | - | - | - |
| P64 | 36 | Male | Influenza virus | <10^3 | 10^5 | Yes |  | Not detected | <10^3 | No |  | Not detected | Decrease | Yes |
|  |  |  | Aspergillus fumigatus | Not detected | 10^3 | No |  | Not detected | <10^3 | No |  | - | - | - |
|  |  |  | Candida albicans | Not detected | 10^3 | No |  | <10^3 | <10^3 | Yes |  | - | - | - |
| P65 | 81 | Female | Pseudomonas aeruginosa | ≥10^6 | ≥10^6 | Yes |  | ≥10^6 | ≥10^6 | Yes |  | No change | No change | Yes |
|  |  |  | Human alphaherpesvirus 1 | 10^4 | Not detected | No |  | Not detected | Not detected | Yes |  | - | - | - |
|  |  |  | Staphylococcus aureus | 10^3 | 10^3 | Yes |  | 10^5 | 10^4 | Yes |  | Increase | Increase | Yes |
|  |  |  | Corynebacterium striatum | 10^3 | 10^3 | Yes |  | 10^3 | 10^3 | Yes |  | No change | No change | Yes |
|  |  |  | Candida parapsilosis | <10^3 | 10^3 | Yes |  | <10^3 | 10^5 | Yes |  | No change | Increase | No |
|  |  |  | Candida albicans | Not detected | Not detected | Yes |  | <10^3 | Not detected | No |  | - | - | - |
| P66 | 56 | Male | Pseudomonas aeruginosa | ≥10^6 | ≥10^6 | Yes |  | ≥10^6 | ≥10^6 | Yes |  | No change | No change | Yes |
|  |  |  | Acinetobacter baumannii | ≥10^6 | Not detected | No |  | ≥10^6 | Not detected | No |  | - | - | - |
|  |  |  | Klebsiella pneumoniae | ≥10^6 | 10^4 | Yes |  | 10^5 | Not detected | No |  | Decrease | Not detected | Yes |
|  |  |  | Corynebacterium striatum | 10^5 | 10^5 | Yes |  | 10^4 | 10^3 | Yes |  | Decrease | Decrease | Yes |
|  |  |  | Stenotrophomonas maltophilia | 10^5 | Not detected | No |  | Not detected | 10^5 | No |  | - | - | - |
|  |  |  | Cytomegalovirus | 10^4 | 10^5 | Yes |  | Not detected | 10^4 | No |  | Not detected | Decrease | Yes |
|  |  |  | Epstein-Barr virus | 10^4 | 10^5 | Yes |  | 10^4 | 10^4 | Yes |  | No change | Decrease | No |
|  |  |  | Candida albicans | 10^5 | Not detected | No |  | 10^5 | Not detected | No |  | - | - | - |
|  |  |  | Enterococcus faecium | 10^4 | Not detected | No |  | 10^5 | Not detected | No |  | - | - | - |
| P67 | 75 | Male | Acinetobacter baumannii | <10^3 | <10^3 | Yes |  | Not detected | Not detected | Yes |  | Not detected | Not detected | Yes |
|  |  |  | Pseudomonas aeruginosa | <10^3 | <10^3 | Yes |  | Not detected | Not detected | Yes |  | Not detected | Not detected | Yes |
|  |  |  | Candida glabrata | Not detected | 10^5 | No |  | <10^3 | 10^4 | Yes |  | - | - | - |
|  |  |  | Candida albicans | Not detected | 10^5 | No |  | Not detected | <10^3 | No |  | - | - | - |
|  |  |  | Haemophilus influenzae | Not detected | 10^5 | No |  | Not detected | <10^3 | No |  | - | - | - |
|  |  |  | Staphylococcus aureus | Not detected | Not detected | Yes |  | <10^3 | 10^3 | Yes |  | - | - | - |
| P68 | 74 | Male | Klebsiella pneumoniae | 10^5 | 10^5 | Yes |  | 10^5 | Not detected | No |  | No change | Not detected | No |
|  |  |  | Epstein-Barr virus | 10^5 | 10^4 | Yes |  | ≥10^6 | <10^3 | Yes |  | Increase | Decrease | No |
|  |  |  | Corynebacterium striatum | 10^5 | 10^4 | Yes |  | ≥10^6 | 10^4 | Yes |  | Increase | No change | No |
|  |  |  | Human parainfluenza virus | <10^3 | ≥10^6 | Yes |  | 10^4 | ≥10^6 | Yes |  | Increase | No change | No |
|  |  |  | Enterococcus faecium | 10^3 | <10^3 | Yes |  | 10^4 | Not detected | No |  | Increase | Not detected | No |
|  |  |  | Staphylococcus aureus | 10^3 | 10^3 | Yes |  | 10^5 | Not detected | No |  | Increase | Not detected | No |
|  |  |  | Acinetobacter baumannii | <10^3 | <10^3 | Yes |  | Not detected | Not detected | Yes |  | Not detected | Not detected | Yes |
|  |  |  | Influenza virus | Not detected | 10^3 | No |  | 10^4 | Not detected | No |  | - | - | - |
|  |  |  | Candida parapsilosis | Not detected | <10^3 | No |  | 10^4 | 10^5 | Yes |  | - | - | - |
| P69 | 87 | Female | Human alphaherpesvirus 1 | ≥10^6 | 10^3 | Yes |  | 10^3 | 10^4 | Yes |  | Decrease | Increase | No |
|  |  |  | Staphylococcus aureus | 10^5 | 10^5 | Yes |  | 10^4 | <10^3 | Yes |  | Decrease | Decrease | Yes |
|  |  |  | Pseudomonas aeruginosa | 10^4 | 10^5 | Yes |  | ≥10^6 | 10^5 | Yes |  | Increase | No change | No |
|  |  |  | Epstein-Barr virus | 10^4 | 10^3 | Yes |  | ≥10^6 | 10^4 | Yes |  | Increase | Increase | Yes |
|  |  |  | Klebsiella pneumoniae | <10^3 | 10^3 | Yes |  | Not detected | <10^3 | No |  | Not detected | Decrease | Yes |
|  |  |  | Corynebacterium striatum | <10^3 | <10^3 | Yes |  | 10^5 | Not detected | No |  | Increase | Not detected | No |
|  |  |  | Candida glabrata | <10^3 | 10^3 | Yes |  | 10^4 | <10^3 | Yes |  | Increase | Decrease | No |
|  |  |  | Candida albicans | Not detected | 10^3 | No |  | 10^3 | Not detected | No |  | - | - | - |
|  |  |  | Stenotrophomonas maltophilia | Not detected | Not detected | Yes |  | Not detected | 10^4 | No |  | - | - | - |
| P70 | 81 | Male | Epstein-Barr virus | 10^3 | ≥10^6 | Yes |  | 10^3 | 10^3 | Yes |  | No change | Decrease | No |
|  |  |  | Acinetobacter baumannii | <10^3 | Not detected | No |  | <10^3 | ≥10^6 | Yes |  | - | - | - |
|  |  |  | Candida albicans | <10^3 | ≥10^6 | Yes |  | <10^3 | <10^3 | Yes |  | No change | Decrease | No |
|  |  |  | Klebsiella aerogenes | Not detected | 10^4 | No |  | Not detected | Not detected | Yes |  | - | - | - |
|  |  |  | Klebsiella pneumoniae | Not detected | 10^3 | No |  | Not detected | Not detected | Yes |  | - | - | - |
|  |  |  | Cytomegalovirus | Not detected | 10^3 | No |  | Not detected | <10^3 | No |  | - | - | - |
|  |  |  | Human alphaherpesvirus 1 | Not detected | Not detected | Yes |  | 10^3 | ≥10^6 | Yes |  | - | - | - |
|  |  |  | Pneumocystis jirovecii | Not detected | Not detected | Yes |  | Not detected | <10^3 | No |  | - | - | - |
|  |  |  | Pseudomonas aeruginosa | Not detected | Not detected | Yes |  | Not detected | 10^3 | No |  | - | - | - |
| P71 | 74 | Male | Stenotrophomonas maltophilia | ≥10^6 | ≥10^6 | Yes |  | ≥10^6 | 10^4 | Yes |  | No change | Decrease | No |
|  |  |  | Acinetobacter baumannii | ≥10^6 | 10^5 | Yes |  | ≥10^6 | 10^4 | Yes |  | No change | Decrease | No |
|  |  |  | Escherichia coli | ≥10^6 | ≥10^6 | Yes |  | ≥10^6 | 10^4 | Yes |  | No change | Decrease | No |
|  |  |  | Proteus mirabilis | 10^5 | ≥10^6 | Yes |  | ≥10^6 | ≥10^6 | Yes |  | Increase | No change | No |
|  |  |  | Epstein-Barr virus | 10^5 | 10^4 | Yes |  | Not detected | 10^4 | No |  | Not detected | No change | No |
|  |  |  | Corynebacterium striatum | 10^4 | Not detected | No |  | 10^4 | Not detected | No |  | - | - | - |
|  |  |  | Klebsiella pneumoniae | 10^3 | 10^4 | Yes |  | Not detected | Not detected | Yes |  | Not detected | Not detected | Yes |
|  |  |  | Cytomegalovirus | Not detected | 10^3 | No |  | Not detected | 10^4 | No |  | - | - | - |
|  |  |  | Pseudomonas aeruginosa | Not detected | Not detected | Yes |  | ≥10^6 | ≥10^6 | Yes |  | - | - | - |
|  |  |  | Enterococcus faecium | Not detected | Not detected | Yes |  | <10^3 | ≥10^6 | Yes |  | - | - | - |
|  |  |  | Candida glabrata | Not detected | Not detected | Yes |  | <10^3 | 10^3 | Yes |  | - | - | - |
| P72 | 63 | Male | Streptococcus pneumoniae | ≥10^6 | ≥10^6 | Yes |  | Not detected | Not detected | Yes |  | Not detected | Not detected | Yes |
|  |  |  | Pseudomonas aeruginosa | 10^4 | 10^4 | Yes |  | ≥10^6 | ≥10^6 | Yes |  | Increase | Increase | Yes |
|  |  |  | Mycobacteroides abscessus | 10^3 | 10^3 | Yes |  | 10^3 | 10^4 | Yes |  | No change | Increase | No |
|  |  |  | Corynebacterium striatum | 10^4 | 10^5 | Yes |  | 10^5 | ≥10^6 | Yes |  | Increase | Increase | Yes |
|  |  |  | Elizabethkingia anophelis | Not detected | 10^4 | No |  | 10^4 | 10^4 | Yes |  | - | - | - |
|  |  |  | Candida auris | Not detected | 10^3 | No |  | <10^3 | 10^5 | Yes |  | - | - | - |
|  |  |  | Candida parapsilosis | Not detected | <10^3 | No |  | Not detected | 10^3 | No |  | - | - | - |
|  |  |  | Acinetobacter baumannii | Not detected | Not detected | Yes |  | ≥10^6 | Not detected | No |  | - | - | - |
|  |  |  | Human alphaherpesvirus 1 | Not detected | Not detected | Yes |  | 10^3 | Not detected | No |  | - | - | - |
|  |  |  | Epstein-Barr virus | Not detected | Not detected | Yes |  | 10^3 | Not detected | No |  | - | - | - |
|  |  |  | Cytomegalovirus | Not detected | Not detected | Yes |  | Not detected | <10^3 | No |  | - | - | - |
| P73 | 72 | Female | Human alphaherpesvirus 1 | ≥10^6 | ≥10^6 | Yes |  | - | - | - |  | - | - | - |
|  |  |  | Corynebacterium striatum | 10^4 | 10^3 | Yes |  | - | - | - |  | - | - | - |
|  |  |  | Epstein-Barr virus | 10^3 | 10^5 | Yes |  | - | - | - |  | - | - | - |
|  |  |  | Influenza virus | Not detected | ≥10^6 | No |  | - | - | - |  | - | - | - |
|  |  |  | Klebsiella aerogenes | Not detected | 10^5 | No |  | - | - | - |  | - | - | - |
|  |  |  | Aspergillus flavus | Not detected | 10^3 | No |  | - | - | - |  | - | - | - |
|  |  |  | Acinetobacter baumannii | Not detected | 10^3 | No |  | - | - | - |  | - | - | - |
|  |  |  | Candida parapsilosis | Not detected | 10^3 | No |  | - | - | - |  | - | - | - |
| P74 | 70 | Male | Candida albicans | ≥10^6 | 10^5 | Yes |  | - | - | - |  | - | - | - |
|  |  |  | Epstein-Barr virus | ≥10^6 | 10^3 | Yes |  | - | - | - |  | - | - | - |
|  |  |  | Klebsiella pneumoniae | 10^5 | ≥10^6 | Yes |  | - | - | - |  | - | - | - |
|  |  |  | Human alphaherpesvirus 1 | 10^4 | Not detected | No |  | - | - | - |  | - | - | - |
|  |  |  | Severe acute respiratory syndrome coronavirus 2 | 10^4 | 10^3 | Yes |  | - | - | - |  | - | - | - |
|  |  |  | Enterococcus faecium | ≥10^6 | 10^4 | Yes |  | - | - | - |  | - | - | - |
|  |  |  | Corynebacterium striatum | 10^5 | 10^4 | Yes |  | - | - | - |  | - | - | - |
|  |  |  | Acinetobacter baumannii | 10^3 | 10^3 | Yes |  | - | - | - |  | - | - | - |
|  |  |  | Pneumocystis jirovecii | Not detected | <10^3 | No |  | - | - | - |  | - | - | - |
| P75 | 78 | Female | Human alphaherpesvirus 1 | ≥10^6 | Not detected | No |  | - | - | - |  | - | - | - |
|  |  |  | Pseudomonas aeruginosa | 10^5 | ≥10^6 | Yes |  | - | - | - |  | - | - | - |
|  |  |  | Stenotrophomonas maltophilia | 10^5 | 10^4 | Yes |  | - | - | - |  | - | - | - |
|  |  |  | Serratia marcescens | 10^5 | <10^3 | Yes |  | - | - | - |  | - | - | - |
|  |  |  | Proteus mirabilis | 10^5 | Not detected | No |  | - | - | - |  | - | - | - |
|  |  |  | Achromobacter xylosoxidans | 10^5 | 10^4 | Yes |  | - | - | - |  | - | - | - |
|  |  |  | Corynebacterium striatum | 10^4 | <10^3 | Yes |  | - | - | - |  | - | - | - |
|  |  |  | Epstein-Barr virus | 10^3 | 10^3 | Yes |  | - | - | - |  | - | - | - |
|  |  |  | Acinetobacter baumannii | 10^3 | 10^3 | Yes |  | - | - | - |  | - | - | - |
|  |  |  | Klebsiella pneumoniae | 10^3 | 10^5 | Yes |  | - | - | - |  | - | - | - |
|  |  |  | Elizabethkingia anophelis | <10^3 | 10^3 | Yes |  | - | - | - |  | - | - | - |
| P76 | 86 | Male | Corynebacterium striatum | ≥10^6 | Not detected | No |  | - | - | - |  | - | - | - |
|  |  |  | Enterococcus faecium | 10^4 | <10^3 | Yes |  | - | - | - |  | - | - | - |
|  |  |  | Candida albicans | 10^3 | Not detected | No |  | - | - | - |  | - | - | - |
|  |  |  | Epstein-Barr virus | 10^3 | Not detected | No |  | - | - | - |  | - | - | - |
|  |  |  | Candida glabrata | <10^3 | Not detected | No |  | - | - | - |  | - | - | - |
|  |  |  | Klebsiella pneumoniae | <10^3 | <10^3 | Yes |  | - | - | - |  | - | - | - |
|  |  |  | Elizabethkingia meningoseptica | <10^3 | 10^3 | Yes |  | - | - | - |  | - | - | - |
|  |  |  | Acinetobacter baumannii | Not detected | 10^3 | No |  | - | - | - |  | - | - | - |
| P77 | 69 | Male | Pseudomonas aeruginosa | ≥10^6 | ≥10^6 | Yes |  | - | - | - |  | - | - | - |
|  |  |  | Klebsiella oxytoca | 10^4 | Not detected | No |  | - | - | - |  | - | - | - |
|  |  |  | Aspergillus flavus | <10^3 | Not detected | No |  | - | - | - |  | - | - | - |
|  |  |  | Achromobacter xylosoxidans | 10^5 | 10^5 | Yes |  | - | - | - |  | - | - | - |
|  |  |  | Enterococcus faecium | 10^5 | 10^3 | Yes |  | - | - | - |  | - | - | - |
|  |  |  | Corynebacterium striatum | 10^5 | Not detected | No |  | - | - | - |  | - | - | - |
|  |  |  | Enterococcus faecalis | 10^4 | Not detected | No |  | - | - | - |  | - | - | - |
|  |  |  | Serratia marcescens | 10^3 | ≥10^6 | Yes |  | - | - | - |  | - | - | - |
|  |  |  | Human alphaherpesvirus 1 | 10^3 | 10^3 | Yes |  | - | - | - |  | - | - | - |
|  |  |  | Epstein-Barr virus | <10^3 | 10^3 | Yes |  | - | - | - |  | - | - | - |
|  |  |  | Candida glabrata | <10^3 | Not detected | No |  | - | - | - |  | - | - | - |
| P78 | 74 | Male | Epstein-Barr virus | 10^4 | 10^4 | Yes |  | Not detected | 10^3 | No |  | Not detected | Decrease | Yes |
|  |  |  | Rhinovirus | 10^4 | 10^3 | Yes |  | 10^3 | 10^3 | Yes |  | Decrease | No change | No |
|  |  |  | Corynebacterium striatum | 10^3 | 10^4 | Yes |  | 10^4 | 10^4 | Yes |  | Increase | No change | No |
|  |  |  | Cytomegalovirus | <10^3 | 10^3 | Yes |  | <10^3 | 10^4 | Yes |  | No change | Increase | No |
|  |  |  | Acinetobacter baumannii | <10^3 | Not detected | No |  | Not detected | Not detected | Yes |  | - | - | - |
|  |  |  | Candida albicans | Not detected | Not detected | Yes |  | Not detected | 10^4 | No |  | - | - | - |
| P79 | 68 | Female | Acinetobacter baumannii | 10^5 | Not detected | No |  | ≥10^6 | Not detected | No |  | - | - | - |
|  |  |  | Achromobacter xylosoxidans | 10^4 | ≥10^6 | Yes |  | <10^3 | 10^4 | Yes |  | Decrease | Decrease | Yes |
|  |  |  | Elizabethkingia anophelis | 10^3 | ≥10^6 | Yes |  | <10^3 | 10^5 | Yes |  | Decrease | Decrease | Yes |
|  |  |  | Corynebacterium striatum | 10^3 | 10^5 | Yes |  | 10^4 | 10^5 | Yes |  | Increase | No change | No |
|  |  |  | Pseudomonas aeruginosa | <10^3 | 10^5 | Yes |  | <10^3 | 10^4 | Yes |  | No change | Decrease | No |
|  |  |  | Candida albicans | <10^3 | 10^3 | Yes |  | 10^4 | Not detected | No |  | Increase | Not detected | No |
|  |  |  | Stenotrophomonas maltophilia | Not detected | ≥10^6 | No |  | Not detected | Not detected | Yes |  | - | - | - |
|  |  |  | Elizabethkingia meningoseptica | Not detected | ≥10^6 | No |  | Not detected | 10^3 | No |  | - | - | - |
|  |  |  | Candida parapsilosis | Not detected | 10^5 | No |  | Not detected | 10^3 | No |  | - | - | - |
|  |  |  | Mycobacteroides abscessus | Not detected | 10^3 | No |  | Not detected | Not detected | Yes |  | - | - | - |
|  |  |  | Cytomegalovirus | Not detected | 10^3 | No |  | <10^3 | 10^3 | Yes |  | - | - | - |
|  |  |  | Klebsiella pneumoniae | Not detected | 10^3 | No |  | Not detected | Not detected | Yes |  | - | - | - |
|  |  |  | Epstein-Barr virus | Not detected | Not detected | Yes |  | 10^3 | 10^3 | Yes |  | - | - | - |
| P80 | 64 | Male | Corynebacterium striatum | 10^5 | ≥10^6 | Yes |  | - | - | - |  | - | - | - |
|  |  |  | Stenotrophomonas maltophilia | 10^5 | 10^5 | Yes |  | - | - | - |  | - | - | - |
|  |  |  | Klebsiella pneumoniae | 10^4 | <10^3 | Yes |  | - | - | - |  | - | - | - |
|  |  |  | Influenza virus | <10^3 | <10^3 | Yes |  | - | - | - |  | - | - | - |
|  |  |  | Enterococcus faecium | 10^3 | ≥10^6 | Yes |  | - | - | - |  | - | - | - |
|  |  |  | Cytomegalovirus | 10^3 | 10^5 | Yes |  | - | - | - |  | - | - | - |
|  |  |  | Elizabethkingia anophelis | 10^3 | 10^3 | Yes |  | - | - | - |  | - | - | - |
|  |  |  | Aspergillus fumigatus | Not detected | 10^3 | No |  | - | - | - |  | - | - | - |
| P81 | 76 | Male | Corynebacterium striatum | 10^5 | 10^5 | Yes |  | 10^5 | 10^3 | Yes |  | No change | Decrease | No |
|  |  |  | Enterococcus faecium | 10^3 | 10^5 | Yes |  | 10^4 | 10^4 | Yes |  | Increase | Decrease | No |
|  |  |  | Klebsiella pneumoniae | 10^3 | ≥10^6 | Yes |  | 10^4 | ≥10^6 | Yes |  | Increase | No change | No |
|  |  |  | Acinetobacter baumannii | <10^3 | 10^5 | Yes |  | Not detected | Not detected | Yes |  | Not detected | Not detected | Yes |
|  |  |  | Candida tropicalis | <10^3 | 10^5 | Yes |  | Not detected | <10^3 | No |  | Not detected | Decrease | Yes |
|  |  |  | Candida albicans | Not detected | 10^5 | No |  | 10^3 | 10^3 | Yes |  | - | - | - |
|  |  |  | Stenotrophomonas maltophilia | Not detected | 10^4 | No |  | 10^3 | 10^5 | Yes |  | - | - | - |
|  |  |  | Epstein-Barr virus | Not detected | Not detected | Yes |  | 10^3 | 10^3 | Yes |  | - | - | - |
|  |  |  | Elizabethkingia anophelis | Not detected | Not detected | Yes |  | Not detected | 10^4 | No |  | - | - | - |
| P82 | 88 | Female | Acinetobacter baumannii | ≥10^6 | ≥10^6 | Yes |  | 10^4 | <10^3 | Yes |  | Decrease | Decrease | Yes |
|  |  |  | Stenotrophomonas maltophilia | ≥10^6 | 10^5 | Yes |  | 10^3 | 10^5 | Yes |  | Decrease | No change | No |
|  |  |  | Corynebacterium striatum | ≥10^6 | ≥10^6 | Yes |  | ≥10^6 | ≥10^6 | Yes |  | No change | No change | Yes |
|  |  |  | Epstein-Barr virus | ≥10^6 | 10^4 | Yes |  | <10^3 | ≥10^6 | Yes |  | Decrease | Increase | No |
|  |  |  | Klebsiella pneumoniae | 10^5 | ≥10^6 | Yes |  | 10^3 | <10^3 | Yes |  | Decrease | Decrease | Yes |
|  |  |  | Candida albicans | 10^4 | 10^3 | Yes |  | <10^3 | 10^4 | Yes |  | Decrease | Increase | No |
|  |  |  | Enterococcus faecalis | 10^3 | 10^3 | Yes |  | Not detected | Not detected | Yes |  | Not detected | Not detected | Yes |
|  |  |  | Staphylococcus aureus | Not detected | 10^4 | No |  | 10^5 | 10^3 | Yes |  | - | - | - |
|  |  |  | Candida parapsilosis | Not detected | Not detected | Yes |  | 10^4 | 10^3 | Yes |  | - | - | - |
|  |  |  | Candida glabrata | Not detected | Not detected | Yes |  | Not detected | 10^4 | No |  | - | - | - |
| P83 | 67 | Female | Varicella-zoster virus | ≥10^6 | ≥10^6 | Yes |  | 10^5 | ≥10^6 | Yes |  | Decrease | No change | No |
|  |  |  | Acinetobacter baumannii | <10^3 | 10^5 | Yes |  | Not detected | Not detected | Yes |  | Not detected | Not detected | Yes |
|  |  |  | Human alphaherpesvirus 1 | Not detected | 10^4 | No |  | Not detected | Not detected | Yes |  | - | - | - |
|  |  |  | Pseudomonas aeruginosa | Not detected | 10^4 | No |  | Not detected | Not detected | Yes |  | - | - | - |
|  |  |  | Epstein-Barr virus | Not detected | 10^3 | No |  | Not detected | Not detected | Yes |  | - | - | - |
|  |  |  | Stenotrophomonas maltophilia | Not detected | Not detected | Yes |  | ≥10^6 | ≥10^6 | Yes |  | - | - | - |
|  |  |  | Corynebacterium striatum | Not detected | Not detected | Yes |  | ≥10^6 | ≥10^6 | Yes |  | - | - | - |
|  |  |  | Staphylococcus aureus | Not detected | Not detected | Yes |  | 10^5 | 10^5 | Yes |  | - | - | - |
| P84 | 71 | Male | Epstein-Barr virus | 10^3 | 10^3 | Yes |  | - | - | - |  | - | - | - |
|  |  |  | Candida parapsilosis | <10^3 | Not detected | No |  | - | - | - |  | - | - | - |
|  |  |  | Corynebacterium striatum | Not detected | <10^3 | No |  | - | - | - |  | - | - | - |
|  |  |  | Human alphaherpesvirus 1 | Not detected | <10^3 | No |  | - | - | - |  | - | - | - |
| P85 | 70 | Male | Candida albicans | ≥10^6 | 10^4 | Yes |  | 10^3 | 10^3 | Yes |  | Decrease | Decrease | Yes |
|  |  |  | Pseudomonas aeruginosa | 10^4 | 10^5 | Yes |  | ≥10^6 | ≥10^6 | Yes |  | Increase | Increase | Yes |
|  |  |  | Staphylococcus aureus | 10^4 | 10^4 | Yes |  | Not detected | Not detected | Yes |  | Not detected | Not detected | Yes |
|  |  |  | Klebsiella pneumoniae | 10^3 | <10^3 | Yes |  | 10^3 | 10^3 | Yes |  | No change | Increase | No |
|  |  |  | Candida parapsilosis | <10^3 | Not detected | No |  | Not detected | Not detected | Yes |  | - | - | - |
|  |  |  | Pneumocystis jirovecii | <10^3 | <10^3 | Yes |  | Not detected | Not detected | Yes |  | Not detected | Not detected | Yes |
|  |  |  | Corynebacterium striatum | Not detected | Not detected | Yes |  | 10^5 | 10^5 | Yes |  | - | - | - |
|  |  |  | Stenotrophomonas maltophilia | Not detected | Not detected | Yes |  | 10^4 | 10^4 | Yes |  | - | - | - |
|  |  |  | Epstein-Barr virus | Not detected | Not detected | Yes |  | Not detected | 10^3 | No |  | - | - | - |
| P86 | 96 | Female | Candida tropicalis | ≥10^6 | ≥10^6 | Yes |  | - | - | - |  | - | - | - |
|  |  |  | Candida parapsilosis | 10^5 | 10^3 | Yes |  | - | - | - |  | - | - | - |
|  |  |  | Enterococcus faecalis | 10^3 | 10^3 | Yes |  | - | - | - |  | - | - | - |
|  |  |  | Acinetobacter baumannii | 10^3 | 10^3 | Yes |  | - | - | - |  | - | - | - |
| P87 | 65 | Male | Stenotrophomonas maltophilia | ≥10^6 | 10^3 | Yes |  | 10^5 | 10^3 | Yes |  | Decrease | No change | No |
|  |  |  | Pseudomonas aeruginosa | ≥10^6 | ≥10^6 | Yes |  | ≥10^6 | ≥10^6 | Yes |  | No change | No change | Yes |
|  |  |  | Proteus mirabilis | ≥10^6 | <10^3 | Yes |  | 10^5 | <10^3 | Yes |  | Decrease | No change | No |
|  |  |  | Corynebacterium striatum | ≥10^6 | 10^5 | Yes |  | 10^3 | ≥10^6 | Yes |  | Decrease | Increase | No |
|  |  |  | Achromobacter xylosoxidans | 10^4 | 10^3 | Yes |  | Not detected | Not detected | Yes |  | Not detected | Not detected | Yes |
|  |  |  | Candida glabrata | 10^3 | 10^4 | Yes |  | 10^3 | Not detected | No |  | No change | Not detected | No |
|  |  |  | Enterococcus faecium | 10^3 | Not detected | No |  | 10^3 | <10^3 | Yes |  | - | - | - |
|  |  |  | Candida albicans | 10^3 | 10^4 | Yes |  | <10^3 | Not detected | No |  | Decrease | Not detected | Yes |
|  |  |  | Klebsiella pneumoniae | <10^3 | 10^4 | Yes |  | 10^5 | 10^4 | Yes |  | Increase | No change | No |
|  |  |  | Human alphaherpesvirus 1 | <10^3 | <10^3 | Yes |  | Not detected | <10^3 | No |  | Not detected | No change | No |
|  |  |  | Elizabethkingia anophelis | Not detected | <10^3 | No |  | Not detected | Not detected | Yes |  | - | - | - |
|  |  |  | Serratia marcescens | Not detected | Not detected | Yes |  | 10^5 | Not detected | No |  | - | - | - |
|  |  |  | Epstein-Barr virus | Not detected | Not detected | Yes |  | <10^3 | <10^3 | Yes |  | - | - | - |
|  |  |  | Acinetobacter baumannii | Not detected | Not detected | Yes |  | Not detected | 10^3 | No |  | - | - | - |
| P88 | 76 | Female | Escherichia coli | 10^5 | Not detected | No |  | - | - | - |  | - | - | - |
|  |  |  | Acinetobacter baumannii | 10^4 | ≥10^6 | Yes |  | - | - | - |  | - | - | - |
|  |  |  | Candida albicans | 10^4 | Not detected | No |  | - | - | - |  | - | - | - |
|  |  |  | Candida parapsilosis | 10^4 | Not detected | No |  | - | - | - |  | - | - | - |
|  |  |  | Candida tropicalis | <10^3 | Not detected | No |  | - | - | - |  | - | - | - |
|  |  |  | Enterococcus faecium | <10^3 | 10^3 | Yes |  | - | - | - |  | - | - | - |
|  |  |  | Human alphaherpesvirus 1 | <10^3 | Not detected | No |  | - | - | - |  | - | - | - |
|  |  |  | Mycobacterium intracellulare | Not detected | 10^5 | No |  | - | - | - |  | - | - | - |
|  |  |  | Aspergillus fumigatus | Not detected | 10^4 | No |  | - | - | - |  | - | - | - |
|  |  |  | Nocardia cyriacigeorgica | Not detected | 10^3 | No |  | - | - | - |  | - | - | - |
|  |  |  | Epstein-Barr virus | Not detected | 10^3 | No |  | - | - | - |  | - | - | - |
| P89 | 84 | Female | Pseudomonas aeruginosa | ≥10^6 | ≥10^6 | Yes |  | ≥10^6 | ≥10^6 | Yes |  | No change | No change | Yes |
|  |  |  | Corynebacterium striatum | ≥10^6 | ≥10^6 | Yes |  | ≥10^6 | ≥10^6 | Yes |  | No change | No change | Yes |
|  |  |  | Enterococcus faecium | 10^3 | <10^3 | Yes |  | 10^3 | Not detected | No |  | No change | Not detected | No |
|  |  |  | Klebsiella pneumoniae | 10^3 | Not detected | No |  | 10^3 | Not detected | No |  | - | - | - |
|  |  |  | Cytomegalovirus | 10^3 | 10^3 | Yes |  | 10^4 | Not detected | No |  | Increase | Not detected | No |
|  |  |  | Elizabethkingia anophelis | 10^3 | ≥10^6 | Yes |  | 10^5 | ≥10^6 | Yes |  | Increase | No change | No |
|  |  |  | Epstein-Barr virus | 10^3 | Not detected | No |  | Not detected | Not detected | Yes |  | - | - | - |
|  |  |  | Candida glabrata | <10^3 | Not detected | No |  | 10^3 | Not detected | No |  | - | - | - |
|  |  |  | Candida albicans | <10^3 | 10^3 | Yes |  | 10^4 | 10^5 | Yes |  | Increase | Increase | Yes |
|  |  |  | Mycobacterium intracellulare | Not detected | <10^3 | No |  | Not detected | Not detected | Yes |  | - | - | - |
|  |  |  | Staphylococcus aureus | Not detected | Not detected | Yes |  | 10^5 | Not detected | No |  | - | - | - |
|  |  |  | Achromobacter xylosoxidans | Not detected | Not detected | Yes |  | 10^5 | 10^4 | Yes |  | - | - | - |
|  |  |  | Candida auris | Not detected | Not detected | Yes |  | Not detected | <10^3 | No |  | - | - | - |
| P90 | 90 | Female | Klebsiella pneumoniae | ≥10^6 | ≥10^6 | Yes |  | ≥10^6 | ≥10^6 | Yes |  | No change | No change | Yes |
|  |  |  | Epstein-Barr virus | ≥10^6 | 10^3 | Yes |  | ≥10^6 | 10^4 | Yes |  | No change | Increase | No |
|  |  |  | Corynebacterium striatum | 10^5 | 10^5 | Yes |  | 10^5 | 10^5 | Yes |  | No change | No change | Yes |
|  |  |  | Stenotrophomonas maltophilia | 10^4 | 10^5 | Yes |  | 10^5 | 10^3 | Yes |  | Increase | Decrease | No |
|  |  |  | Elizabethkingia anophelis | 10^3 | <10^3 | Yes |  | 10^3 | <10^3 | Yes |  | No change | No change | Yes |
|  |  |  | Candida albicans | <10^3 | 10^5 | Yes |  | 10^3 | Not detected | No |  | Increase | Not detected | No |
|  |  |  | Candida parapsilosis | Not detected | <10^3 | No |  | Not detected | Not detected | Yes |  | - | - | - |
|  |  |  | Human alphaherpesvirus 1 | Not detected | Not detected | Yes |  | <10^3 | <10^3 | Yes |  | - | - | - |
|  |  |  | Haemophilus influenzae | Not detected | Not detected | Yes |  | Not detected | 10^4 | No |  | - | - | - |
|  |  |  | Cytomegalovirus | Not detected | Not detected | Yes |  | Not detected | <10^3 | No |  | - | - | - |
| P91 | 69 | Female | Acinetobacter baumannii | ≥10^6 | ≥10^6 | Yes |  | 10^4 | 10^5 | Yes |  | Decrease | Decrease | Yes |
|  |  |  | Corynebacterium striatum | ≥10^6 | 10^3 | Yes |  | ≥10^6 | 10^5 | Yes |  | No change | Increase | No |
|  |  |  | Candida parapsilosis | ≥10^6 | Not detected | No |  | 10^4 | Not detected | No |  | - | - | - |
|  |  |  | Staphylococcus aureus | ≥10^6 | <10^3 | Yes |  | 10^4 | Not detected | No |  | Decrease | Not detected | Yes |
|  |  |  | Proteus mirabilis | 10^5 | ≥10^6 | Yes |  | 10^3 | Not detected | No |  | Decrease | Not detected | Yes |
|  |  |  | Epstein-Barr virus | 10^3 | 10^5 | Yes |  | 10^4 | 10^5 | Yes |  | Increase | No change | No |
|  |  |  | Escherichia coli | 10^3 | ≥10^6 | Yes |  | Not detected | Not detected | Yes |  | Not detected | Not detected | Yes |
|  |  |  | Klebsiella pneumoniae | 10^3 | 10^5 | Yes |  | 10^4 | ≥10^6 | Yes |  | Increase | Increase | Yes |
|  |  |  | Candida albicans | 10^3 | Not detected | No |  | 10^4 | 10^3 | Yes |  | - | - | - |
|  |  |  | Enterococcus faecalis | <10^3 | Not detected | No |  | Not detected | Not detected | Yes |  | - | - | - |
|  |  |  | Morganella morganii | <10^3 | 10^3 | Yes |  | <10^3 | 10^4 | Yes |  | No change | Increase | No |
|  |  |  | Cytomegalovirus | <10^3 | Not detected | No |  | 10^5 | Not detected | No |  | - | - | - |
|  |  |  | Human alphaherpesvirus 1 | <10^3 | <10^3 | Yes |  | 10^4 | 10^3 | Yes |  | Increase | Increase | Yes |
|  |  |  | Pseudomonas aeruginosa | Not detected | Not detected | Yes |  | ≥10^6 | ≥10^6 | Yes |  | - | - | - |
| P92 | 69 | Male | Pseudomonas aeruginosa | ≥10^6 | ≥10^6 | Yes |  | ≥10^6 | ≥10^6 | Yes |  | No change | No change | Yes |
|  |  |  | Corynebacterium striatum | 10^4 | 10^3 | Yes |  | 10^4 | 10^4 | Yes |  | No change | Increase | No |
|  |  |  | Epstein-Barr virus | 10^3 | ≥10^6 | Yes |  | 10^3 | 10^4 | Yes |  | No change | Decrease | No |
|  |  |  | Stenotrophomonas maltophilia | 10^3 | ≥10^6 | Yes |  | 10^5 | ≥10^6 | Yes |  | Increase | No change | No |
|  |  |  | Candida albicans | Not detected | 10^3 | No |  | 10^3 | 10^3 | Yes |  | - | - | - |
|  |  |  | Elizabethkingia meningoseptica | Not detected | 10^3 | No |  | <10^3 | <10^3 | Yes |  | - | - | - |
|  |  |  | Candida tropicalis | Not detected | 10^3 | No |  | 10^3 | 10^3 | Yes |  | - | - | - |
|  |  |  | Klebsiella pneumoniae | Not detected | <10^3 | No |  | Not detected | Not detected | Yes |  | - | - | - |
|  |  |  | Enterococcus faecium | Not detected | Not detected | Yes |  | <10^3 | <10^3 | Yes |  | - | - | - |
|  |  |  | Candida parapsilosis | Not detected | Not detected | Yes |  | Not detected | <10^3 | No |  | - | - | - |
| P93 | 89 | Female | Staphylococcus aureus | ≥10^6 | ≥10^6 | Yes |  | 10^5 | Not detected | No |  | Decrease | Not detected | Yes |
|  |  |  | Epstein-Barr virus | 10^3 | <10^3 | Yes |  | Not detected | <10^3 | No |  | Not detected | No change | No |
|  |  |  | Candida albicans | 10^3 | ≥10^6 | Yes |  | ≥10^6 | ≥10^6 | Yes |  | Increase | No change | No |
|  |  |  | Enterococcus faecium | <10^3 | Not detected | No |  | Not detected | Not detected | Yes |  | - | - | - |
|  |  |  | Human alphaherpesvirus 1 | Not detected | ≥10^6 | No |  | Not detected | Not detected | Yes |  | - | - | - |
|  |  |  | Pneumocystis jirovecii | Not detected | <10^3 | No |  | Not detected | Not detected | Yes |  | - | - | - |
|  |  |  | Pseudomonas aeruginosa | Not detected | 10^3 | No |  | ≥10^6 | <10^3 | Yes |  | - | - | - |
|  |  |  | Cytomegalovirus | Not detected | <10^3 | No |  | Not detected | <10^3 | No |  | - | - | - |
|  |  |  | Aspergillus flavus | Not detected | Not detected | Yes |  | 10^4 | Not detected | No |  | - | - | - |
|  |  |  | Enterococcus faecalis | Not detected | Not detected | Yes |  | 10^3 | Not detected | No |  | - | - | - |
| P94 | 90 | Female | Human alphaherpesvirus 1 | 10^5 | <10^3 | Yes |  | Not detected | 10^3 | No |  | Not detected | Increase | No |
|  |  |  | Corynebacterium striatum | 10^5 | 10^4 | Yes |  | 10^5 | <10^3 | Yes |  | No change | Decrease | No |
|  |  |  | Candida albicans | 10^4 | 10^4 | Yes |  | 10^3 | <10^3 | Yes |  | Decrease | Decrease | Yes |
|  |  |  | Enterococcus faecium | 10^4 | 10^4 | Yes |  | 10^3 | 10^3 | Yes |  | Decrease | Decrease | Yes |
|  |  |  | Acinetobacter baumannii | 10^4 | 10^5 | Yes |  | ≥10^6 | ≥10^6 | Yes |  | Increase | Increase | Yes |
|  |  |  | Epstein-Barr virus | 10^3 | <10^3 | Yes |  | Not detected | <10^3 | No |  | Not detected | No change | No |
|  |  |  | Staphylococcus aureus | <10^3 | 10^3 | Yes |  | 10^3 | Not detected | No |  | Increase | Not detected | No |
|  |  |  | Cytomegalovirus | <10^3 | 10^3 | Yes |  | 10^3 | <10^3 | Yes |  | Increase | Decrease | No |
|  |  |  | Klebsiella pneumoniae | Not detected | Not detected | Yes |  | Not detected | 10^5 | No |  | - | - | - |
|  |  |  | Pseudomonas aeruginosa | Not detected | Not detected | Yes |  | Not detected | 10^3 | No |  | - | - | - |
|  |  |  | Stenotrophomonas maltophilia | Not detected | Not detected | Yes |  | Not detected | 10^3 | No |  | - | - | - |
| P95 | 83 | Male | Pseudomonas aeruginosa | ≥10^6 | ≥10^6 | Yes |  | ≥10^6 | ≥10^6 | Yes |  | No change | No change | Yes |
|  |  |  | Corynebacterium striatum | 10^4 | 10^4 | Yes |  | 10^5 | 10^4 | Yes |  | Increase | No change | No |
|  |  |  | Streptococcus pneumoniae | 10^4 | ≥10^6 | Yes |  | Not detected | ≥10^6 | No |  | Not detected | No change | No |
|  |  |  | Serratia marcescens | 10^4 | 10^4 | Yes |  | ≥10^6 | 10^4 | Yes |  | Increase | No change | No |
|  |  |  | Stenotrophomonas maltophilia | <10^3 | 10^4 | Yes |  | Not detected | 10^3 | No |  | Not detected | Decrease | Yes |
|  |  |  | Candida albicans | <10^3 | Not detected | No |  | Not detected | Not detected | Yes |  | - | - | - |
|  |  |  | Acinetobacter baumannii | Not detected | 10^4 | No |  | Not detected | Not detected | Yes |  | - | - | - |
|  |  |  | Cytomegalovirus | Not detected | 10^4 | No |  | Not detected | Not detected | Yes |  | - | - | - |
|  |  |  | Epstein-Barr virus | Not detected | 10^3 | No |  | Not detected | Not detected | Yes |  | - | - | - |
|  |  |  | Elizabethkingia anophelis | Not detected | Not detected | Yes |  | 10^4 | 10^5 | Yes |  | - | - | - |
|  |  |  | Klebsiella pneumoniae | Not detected | Not detected | Yes |  | 10^3 | 10^5 | Yes |  | - | - | - |
|  |  |  | Escherichia coli | Not detected | Not detected | Yes |  | 10^3 | ≥10^6 | Yes |  | - | - | - |
| P96 | 70 | Female | Acinetobacter baumannii | ≥10^6 | ≥10^6 | Yes |  | ≥10^6 | 10^5 | Yes |  | No change | Decrease | No |
|  |  |  | Stenotrophomonas maltophilia | 10^5 | 10^5 | Yes |  | ≥10^6 | 10^3 | Yes |  | Increase | Decrease | No |
|  |  |  | Elizabethkingia anophelis | 10^3 | 10^4 | Yes |  | 10^3 | Not detected | No |  | No change | Not detected | No |
|  |  |  | Corynebacterium striatum | 10^3 | 10^4 | Yes |  | Not detected | Not detected | Yes |  | Not detected | Not detected | Yes |
|  |  |  | Pseudomonas aeruginosa | 10^3 | ≥10^6 | Yes |  | 10^3 | ≥10^6 | Yes |  | No change | No change | Yes |
|  |  |  | Klebsiella pneumoniae | Not detected | ≥10^6 | No |  | 10^5 | Not detected | No |  | - | - | - |
|  |  |  | Serratia marcescens | Not detected | Not detected | Yes |  | ≥10^6 | ≥10^6 | Yes |  | - | - | - |
|  |  |  | Cytomegalovirus | Not detected | Not detected | Yes |  | 10^3 | Not detected | No |  | - | - | - |
| P97 | 89 | Male | Pseudomonas aeruginosa | ≥10^6 | 10^4 | Yes |  | ≥10^6 | 10^5 | Yes |  | No change | Increase | No |
|  |  |  | Acinetobacter baumannii | ≥10^6 | ≥10^6 | Yes |  | ≥10^6 | 10^4 | Yes |  | No change | Decrease | No |
|  |  |  | Corynebacterium striatum | 10^5 | 10^3 | Yes |  | 10^4 | Not detected | No |  | Decrease | Not detected | Yes |
|  |  |  | Klebsiella pneumoniae | 10^4 | ≥10^6 | Yes |  | 10^4 | ≥10^6 | Yes |  | No change | No change | Yes |
|  |  |  | Cytomegalovirus | 10^3 | 10^3 | Yes |  | Not detected | Not detected | Yes |  | Not detected | Not detected | Yes |
|  |  |  | Candida tropicalis | 10^3 | Not detected | No |  | 10^3 | Not detected | No |  | - | - | - |
|  |  |  | Candida albicans | 10^3 | Not detected | No |  | Not detected | Not detected | Yes |  | - | - | - |
|  |  |  | Epstein-Barr virus | <10^3 | 10^4 | Yes |  | Not detected | 10^3 | No |  | Not detected | Decrease | Yes |
|  |  |  | Elizabethkingia anophelis | Not detected | 10^4 | No |  | 10^4 | 10^5 | Yes |  | - | - | - |
|  |  |  | Stenotrophomonas maltophilia | Not detected | 10^4 | No |  | ≥10^6 | 10^4 | Yes |  | - | - | - |
|  |  |  | Human alphaherpesvirus 1 | Not detected | 10^3 | No |  | 10^3 | 10^4 | Yes |  | - | - | - |
|  |  |  | Enterococcus faecium | Not detected | <10^3 | No |  | Not detected | 10^4 | No |  | - | - | - |
|  |  |  | Candida glabrata | Not detected | <10^3 | No |  | Not detected | 10^3 | No |  | - | - | - |
| P98 | 61 | Male | Corynebacterium striatum | ≥10^6 | 10^5 | Yes |  | - | - | - |  | - | - | - |
|  |  |  | Human alphaherpesvirus 1 | ≥10^6 | 10^5 | Yes |  | - | - | - |  | - | - | - |
|  |  |  | Candida parapsilosis | ≥10^6 | <10^3 | Yes |  | - | - | - |  | - | - | - |
|  |  |  | Elizabethkingia anophelis | 10^5 | ≥10^6 | Yes |  | - | - | - |  | - | - | - |
|  |  |  | Enterococcus faecalis | 10^5 | 10^3 | Yes |  | - | - | - |  | - | - | - |
|  |  |  | Stenotrophomonas maltophilia | 10^5 | ≥10^6 | Yes |  | - | - | - |  | - | - | - |
|  |  |  | Staphylococcus aureus | 10^4 | 10^4 | Yes |  | - | - | - |  | - | - | - |
|  |  |  | Cytomegalovirus | <10^3 | 10^3 | Yes |  | - | - | - |  | - | - | - |
| P99 | 89 | Female | Escherichia coli | ≥10^6 | ≥10^6 | Yes |  | ≥10^6 | ≥10^6 | Yes |  | No change | No change | Yes |
|  |  |  | Klebsiella pneumoniae | 10^5 | 10^5 | Yes |  | 10^4 | 10^5 | Yes |  | Decrease | No change | No |
|  |  |  | Candida glabrata | 10^3 | 10^5 | Yes |  | 10^3 | Not detected | No |  | No change | Not detected | No |
|  |  |  | Candida parapsilosis | <10^3 | Not detected | No |  | Not detected | Not detected | Yes |  | - | - | - |
|  |  |  | Human alphaherpesvirus 1 | Not detected | Not detected | Yes |  | ≥10^6 | ≥10^6 | Yes |  | - | - | - |
|  |  |  | Corynebacterium striatum | Not detected | Not detected | Yes |  | 10^5 | 10^4 | Yes |  | - | - | - |
| P100 | 50 | Male | Acinetobacter baumannii | ≥10^6 | 10^5 | Yes |  | - | - | - |  | - | - | - |
|  |  |  | Epstein-Barr virus | 10^5 | 10^5 | Yes |  | - | - | - |  | - | - | - |
|  |  |  | Enterococcus faecium | 10^5 | 10^4 | Yes |  | - | - | - |  | - | - | - |
|  |  |  | Severe acute respiratory syndrome coronavirus 2 | 10^5 | ≥10^6 | Yes |  | - | - | - |  | - | - | - |
|  |  |  | Aspergillus flavus | 10^4 | Not detected | No |  | - | - | - |  | - | - | - |
|  |  |  | Cytomegalovirus | 10^3 | 10^3 | Yes |  | - | - | - |  | - | - | - |
|  |  |  | Human alphaherpesvirus 1 | Not detected | <10^3 | No |  | - | - | - |  | - | - | - |
| P101 | 86 | Male | Staphylococcus aureus | ≥10^6 | 10^5 | Yes |  | - | - | - |  | - | - | - |
|  |  |  | Corynebacterium striatum | ≥10^6 | ≥10^6 | Yes |  | - | - | - |  | - | - | - |
|  |  |  | Enterococcus faecium | 10^5 | 10^4 | Yes |  | - | - | - |  | - | - | - |
|  |  |  | Epstein-Barr virus | 10^4 | 10^4 | Yes |  | - | - | - |  | - | - | - |
|  |  |  | Enterococcus faecalis | 10^3 | 10^4 | Yes |  | - | - | - |  | - | - | - |
|  |  |  | Candida albicans | <10^3 | 10^5 | Yes |  | - | - | - |  | - | - | - |
|  |  |  | Cytomegalovirus | Not detected | 10^3 | No |  | - | - | - |  | - | - | - |
| P102 | 92 | Male | Corynebacterium striatum | ≥10^6 | ≥10^6 | Yes |  | - | - | - |  | - | - | - |
|  |  |  | Candida parapsilosis | 10^4 | 10^3 | Yes |  | - | - | - |  | - | - | - |
|  |  |  | Epstein-Barr virus | 10^4 | 10^5 | Yes |  | - | - | - |  | - | - | - |
|  |  |  | Enterococcus faecium | 10^3 | ≥10^6 | Yes |  | - | - | - |  | - | - | - |
|  |  |  | Cytomegalovirus | <10^3 | <10^3 | Yes |  | - | - | - |  | - | - | - |
|  |  |  | Human parainfluenza virus | Not detected | 10^4 | No |  | - | - | - |  | - | - | - |
|  |  |  | Candida tropicalis | Not detected | 10^3 | No |  | - | - | - |  | - | - | - |
| P103 | 46 | Female | Escherichia coli | <10^3 | Not detected | No |  | - | - | - |  | - | - | - |
|  |  |  | Haemophilus influenzae | Not detected | 10^5 | No |  | - | - | - |  | - | - | - |
|  |  |  | Human alphaherpesvirus 1 | Not detected | 10^4 | No |  | - | - | - |  | - | - | - |
|  |  |  | Rhinovirus | Not detected | 10^3 | No |  | - | - | - |  | - | - | - |
|  |  |  | Elizabethkingia anophelis | Not detected | <10^3 | No |  | - | - | - |  | - | - | - |
| P104 | 74 | Male | Acinetobacter baumannii | ≥10^6 | 10^4 | Yes |  | 10^3 | Not detected | No |  | Decrease | Not detected | Yes |
|  |  |  | Enterococcus faecium | 10^5 | 10^5 | Yes |  | 10^5 | 10^4 | Yes |  | No change | Decrease | No |
|  |  |  | Klebsiella pneumoniae | 10^4 | 10^4 | Yes |  | 10^3 | 10^5 | Yes |  | Decrease | Increase | No |
|  |  |  | Candida albicans | 10^4 | 10^4 | Yes |  | 10^5 | 10^5 | Yes |  | Increase | Increase | Yes |
|  |  |  | Candida parapsilosis | 10^3 | 10^4 | Yes |  | ≥10^6 | 10^5 | Yes |  | Increase | Increase | Yes |
|  |  |  | Candida glabrata | 10^3 | 10^4 | Yes |  | 10^5 | 10^3 | Yes |  | Increase | Decrease | No |
|  |  |  | Corynebacterium striatum | 10^3 | 10^4 | Yes |  | ≥10^6 | 10^5 | Yes |  | Increase | Increase | Yes |
|  |  |  | Epstein-Barr virus | <10^3 | Not detected | No |  | 10^4 | 10^3 | Yes |  | - | - | - |
|  |  |  | Human parainfluenza virus | Not detected | ≥10^6 | No |  | Not detected | 10^5 | No |  | - | - | - |
|  |  |  | Pseudomonas aeruginosa | Not detected | Not detected | Yes |  | 10^4 | ≥10^6 | Yes |  | - | - | - |
|  |  |  | Chryseobacterium indologenes | Not detected | Not detected | Yes |  | 10^3 | 10^4 | Yes |  | - | - | - |
|  |  |  | Human alphaherpesvirus 1 | Not detected | Not detected | Yes |  | Not detected | <10^3 | No |  | - | - | - |
| P105 | 56 | Male | Acinetobacter baumannii | ≥10^6 | 10^5 | Yes |  | - | - | - |  | - | - | - |
|  |  |  | Klebsiella pneumoniae | 10^4 | 10^3 | Yes |  | - | - | - |  | - | - | - |
|  |  |  | Corynebacterium striatum | 10^4 | 10^4 | Yes |  | - | - | - |  | - | - | - |
|  |  |  | Staphylococcus aureus | 10^4 | Not detected | No |  | - | - | - |  | - | - | - |
|  |  |  | Pseudomonas aeruginosa | <10^3 | 10^4 | Yes |  | - | - | - |  | - | - | - |

| **Table S3. The agreement of all pathogen detection between paired NPS and BALF.** | | | | | | | | |
| --- | --- | --- | --- | --- | --- | --- | --- | --- |
| **Pathogens** | **NPS+/BALF+** | **NPS-/BALF+** | **NPS+/BALF-** | **NPS-/BALF-** | **PPA(%)** | **NPA(%)** | **PPV(%)** | **NPV(%)** |
| *Klebsiella pneumoniae* | 77 | 10 | 10 | 82 | 88.5 | 89.1 | 88.5 | 89.1 |
| *Pseudomonas aeruginosa* | 74 | 10 | 2 | 93 | 88.1 | 97.9 | 97.4 | 90.3 |
| *Stenotrophomonas maltophilia* | 68 | 13 | 3 | 95 | 83.9 | 96.9 | 95.8 | 88.0 |
| *Acinetobacter baumannii* | 60 | 5 | 12 | 102 | 92.3 | 89.5 | 83.3 | 95.3 |
| *Staphylococcus aureus* | 48 | 4 | 20 | 107 | 92.3 | 84.2 | 70.6 | 96.4 |
| *Enterococcus faecium* | 42 | 5 | 20 | 112 | 89.4 | 84.8 | 67.7 | 95.7 |
| *Corynebacterium striatum* | 47 | 1 | 7 | 124 | 97.9 | 94.6 | 87.0 | 99.2 |
| *Achromobacter xylosoxidans* | 23 | 5 | 3 | 148 | 82.1 | 98.0 | 88.5 | 96.7 |
| *Elizabethkingia anophelis* | 19 | 7 | 1 | 152 | 73.1 | 99.3 | 95.0 | 95.6 |
| *Escherichia coli* | 18 | 2 | 11 | 148 | 90.0 | 93.1 | 62.1 | 98.7 |
| *Proteus mirabilis* | 10 | 2 | 6 | 161 | 83.3 | 96.4 | 62.5 | 98.8 |
| *Enterococcus faecalis* | 6 | 1 | 8 | 164 | 85.7 | 95.3 | 42.9 | 99.4 |
| *Serratia marcescens* | 6 | 2 | 2 | 169 | 75.0 | 98.8 | 75.0 | 98.8 |
| *Elizabethkingia meningoseptica* | 4 | 5 | 0 | 170 | 44.4 | 100.0 | 100.0 | 97.1 |
| *Streptococcus pneumoniae* | 6 | 1 | 1 | 171 | 85.7 | 99.4 | 85.7 | 99.4 |
| *Haemophilus influenzae* | 3 | 4 | 0 | 172 | 42.9 | 100.0 | 100.0 | 97.7 |
| *Enterobacter cloacae* | 1 | 2 | 4 | 172 | 33.3 | 97.7 | 20.0 | 98.8 |
| *Mycobacteroides abscessus* | 4 | 1 | 0 | 174 | 80.0 | 100.0 | 100.0 | 99.4 |
| *Streptococcus agalactiae* | 4 | 1 | 0 | 174 | 80.0 | 100.0 | 100.0 | 99.4 |
| *Ureaplasma urealyticum* | 1 | 2 | 0 | 176 | 33.3 | 100.0 | 100.0 | 98.9 |
| *Morganella morganii* | 2 | 0 | 0 | 177 | 100.0 | 100.0 | 100.0 | 100.0 |
| *Mycoplasmoides pneumoniae* | 2 | 0 | 0 | 177 | 100.0 | 100.0 | 100.0 | 100.0 |
| *Klebsiella aerogenes* | 0 | 2 | 0 | 177 | 0.0 | 100.0 | - | 98.9 |
| *Mycobacterium intracellulare* | 0 | 2 | 0 | 177 | 0.0 | 100.0 | - | 98.9 |
| *Chlamydia psittaci* | 1 | 0 | 0 | 178 | 100.0 | 100.0 | 100.0 | 100.0 |
| *Mycobacterium tuberculosis* | 1 | 0 | 0 | 178 | 100.0 | 100.0 | 100.0 | 100.0 |
| *Chryseobacterium indologenes* | 1 | 0 | 0 | 178 | 100.0 | 100.0 | 100.0 | 100.0 |
| *Nocardia cyriacigeorgica* | 0 | 1 | 0 | 178 | 0.0 | 100.0 | - | 99.4 |
| *Klebsiella oxytoca* | 0 | 0 | 1 | 178 | - | 99.4 | 0.0 | 100.0 |
| Epstein-Barr virus | 73 | 13 | 21 | 72 | 84.9 | 77.4 | 77.7 | 84.7 |
| Cytomegalovirus | 27 | 20 | 9 | 123 | 57.4 | 93.2 | 75.0 | 86.0 |
| Human alphaherpesvirus 1 | 30 | 12 | 11 | 126 | 71.4 | 92.0 | 73.2 | 91.3 |
| Influenza virus | 17 | 7 | 1 | 154 | 70.8 | 99.3 | 94.4 | 95.6 |
| Severe acute respiratory syndrome coronavirus 2 | 11 | 0 | 0 | 168 | 100.0 | 100.0 | 100.0 | 100.0 |
| Human respiratory syncytial virus | 4 | 1 | 0 | 174 | 80.0 | 100.0 | 100.0 | 99.4 |
| Human parainfluenza virus | 2 | 3 | 0 | 174 | 40.0 | 100.0 | 100.0 | 98.3 |
| Rhinovirus | 3 | 1 | 0 | 175 | 75.0 | 100.0 | 100.0 | 99.4 |
| Human parvovirus B19 | 2 | 0 | 0 | 177 | 100.0 | 100.0 | 100.0 | 100.0 |
| Varicella-zoster virus | 2 | 0 | 0 | 177 | 100.0 | 100.0 | 100.0 | 100.0 |
| Human metapneumovirus | 1 | 0 | 0 | 178 | 100.0 | 100.0 | 100.0 | 100.0 |
| *Candida albicans* | 43 | 10 | 25 | 101 | 81.1 | 80.1 | 63.2 | 91.0 |
| *Candida parapsilosis* | 14 | 11 | 11 | 143 | 56.0 | 92.8 | 56.0 | 92.8 |
| *Candida glabrata* | 12 | 12 | 11 | 144 | 50.0 | 92.9 | 52.2 | 92.3 |
| *Candida tropicalis* | 10 | 6 | 8 | 155 | 62.5 | 95.1 | 55.6 | 96.3 |
| *Aspergillus fumigatus* | 2 | 8 | 1 | 168 | 20.0 | 99.4 | 66.7 | 95.4 |
| *Candida auris* | 4 | 2 | 4 | 169 | 66.7 | 97.7 | 50.0 | 98.8 |
| *Pneumocystis jirovecii* | 4 | 4 | 0 | 171 | 50.0 | 100.0 | 100.0 | 97.7 |
| *Aspergillus flavus* | 0 | 2 | 3 | 174 | 0.0 | 98.3 | 0.0 | 98.9 |
| Nasopharyngeal swab (NPS), Bronchoalveolar lavage fluid (BALF), Positive percent agreement (PPA), Negative percent agreement (NPA), Positive predictive value (PPV), Negative predictive value (NPV) | | | | | | | | |
